# The Role of Gut Microbiota in Various Neurological and Psychiatric Disorders—An Evidence Mapping Based on Quantified Evidence

**DOI:** 10.1155/2023/5127157

**Published:** 2023-02-08

**Authors:** Yaning Zang, Xigui Lai, Conghui Li, Dongfang Ding, Ying Wang, Yi Zhu

**Affiliations:** ^1^Department of Rehabilitation Medicine, Chengdu Second People's Hospital, Sichuan, China; ^2^Department of Kinesiology, Shanghai University of Sport, Shanghai, China; ^3^The Fifth Affiliated Hospital of Zhengzhou University, Zhengzhou, Henan, China; ^4^Academy of Medical Sciences, Zhengzhou University, Zhengzhou, Henan, China; ^5^The Ninth People's Hospital of Wuxi Affiliated to Soochow University, Wuxi, China

## Abstract

**Methods:**

We searched PubMed, Cochrane Library, and Epistemonikos to identify systematic reviews and meta-analysis (SRs). We searched for neurological diseases and psychiatric disorders, including Alzheimer's disease (AD), attention deficit hyperactivity disorder (ADHD), amyotrophic lateral sclerosis (ALS), autism spectrum disorder (ASD), anorexia nervosa (AN), bipolar disorder (BD), eating disorder (ED), generalized anxiety disorder (GAD), major depressive disorder (MDD), multiple sclerosis (MS), obsessive compulsive disorder (OCD), Parkinson's disease (PD), posttraumatic stress disorder (PTSD), spinal cord injury (SCI), schizophrenia, and stroke. We used A Measurement Tool to Assess Systematic Reviews (AMSTAR-2) to evaluate the quality of included SRs. We also created an evidence map showing the role of gut microbiota in neurological diseases and the certainty of the evidence.

**Results:**

In total, 42 studies were included in this evidence mapping. Most findings were obtained from observational studies. According to the AMSTAR-2 assessment, 21 SRs scored “critically low” in terms of methodological quality, 16 SR scored “low,” and 5 SR scored “moderate.” A total of 15 diseases have been investigated for the potential association between gut microbiome alpha diversity and disease, with the Shannon index and Simpson index being the most widely studied. A total of 12 diseases were investigated for potential link between beta diversity and disease. At the phylum level, *Firmicutes*, *Bacteroidetes*, *Actinobacteria*, *Proteobacteria*, and *Verrucomicrobia* were more researched. At the genus level, *Prevotella*, *Coprococcus*, *Parabacteroides*, *Phascolarctobacterium*, *Escherichia Shigella*, *Alistipes*, *Sutteralla*, *Veillonella*, *Odoribacter*, *Faecalibacterium*, *Bacteroides*, *Bifidobacterium*, *Dialister*, and *Blautia* were more researched. Some diseases have been found to have specific flora changes, and some diseases have been found to have common intestinal microbiological changes.

**Conclusion:**

We found varied levels of evidence for the associations between gut microbiota and neurological diseases; some gut microbiota increased the risk of neurological diseases, whereas others showed evidence of benefit that gut microbiota might be promising therapeutic targets for such diseases.

## 1. Introduction

The intestinal flora mainly exists in the digestive tract and is an important part of the human microbiome. Intestinal flora is characterized by abundant species and large quantity, and the functional potential of different intestinal flora is increasingly understood. Intestinal flora can not only help the body to decompose and store fat but also regulate the immune, endocrine, metabolic, and neurological functions through immune, neuroendocrine, and vagus nerves. Therefore, the occurrence of various diseases of the human body is closely related to the disorder of intestinal flora, such as obesity [1], cardiovascular diseases [2], kidney diseases [3], and nervous system diseases [4].

Studies have found that there are channels in the human body that connect nerves between the gut and the brain, which is known as the microbiota-gut-brain axis [5]. The gut microbiota can regulate neuroinflammation and gastrointestinal symptoms through the gut-brain axis, which has a significant impact on the neurological function of the body not only through the secretion of neurotransmitters but also through immunity and synapses [6]. And, previous studies have shown that inflammatory bowel disease (IBD) patients often suffer from anxiety and depression, which may be associated with impaired brain structure and function and changes in gut microbiome [7–9].

Indeed, since each person features a unique microbiota composition, some systematic review and meta-analysis (SRs) have investigated differences in the composition of the gut microbiota between patients with neurological and psychiatric disorders and healthy individuals [10–12]. For instance, neurogenic bowel dysfunction frequently occurs in patients with spinal cord injury (SCI) and multiple sclerosis (MS) patients who were found to have similar or lower alpha diversity compared to healthy controls [13]. However, SRs tend to focus on specific diseases, such as Parkinson's disease (PD), autism spectrum disorder (ASD), and stroke. To provide an overview of a research area, a novel approach to evidence synthesis research called evidence mapping has been developed [14, 15]. The characteristic of evidence mapping method is that SRs are used as the unit of analysis to extract and classify data, A Measurement Tool to Assess Systematic Reviews (AMSTAR-2) was used to evaluate the credibility of evidence, and it is presented in visual charts.

Therefore, the aim of this evidence mapping was to summarize the gut microbiota associated with neurological and psychiatric disorders and to identify common or differential gut microbiota present in different neurological and psychiatric disorders. Such associations may afford opportunities for both understanding aetiology and making targeted treatment strategies, including probiotic supplements, dietary changes, and even fecal microbial transplants (FMT).

## 2. Methods

### 2.1. Study Design

To summarize the associations of gut microbiota with neurological or psychiatric disorders on a larger scale, we used evidence mapping. This study was conducted on the basis of the methodology proposed by global evidence mapping [16]. The study process was divided into four phases: (1) search strategy and selection, (2) study quality assessment, (3) data extraction, and (4) data synthesis and analysis.

### 2.2. Search Strategy and Selection

A systematic search of the literature was conducted in PubMed, Embase, Cochrane Library, and Epistemonikos databases up to March 21, 2022. Medical subject heading (MeSH) terms and keywords used in the search included various neurological diseases and psychiatric disorders, gastrointestinal microbiome, gut-brain axis, enteric nervous system, and meta-analysis or systematic review. Search results were independently reviewed for eligibility by two independent researchers (Yaning Zang and Ying Wang), with discrepancies resolved by a third researcher (Yi Zhu). Studies were included based on the following criteria (Table 1). Furthermore, the reference list of the relevant reviews has been screened to identify potential studies. Details of the search strategy are provided in Supplementary Material 1.

### 2.3. Study Quality Assessment

The quality of included studies was assessed using the AMSTAR-2 tool, which uses 16 items (critical items: 2, 4, 7, 9, 11, 13, and 15) to assess the methodological quality of systematic reviews or meta-analysis. For each item, there are three answers: yes, partially yes, no. Studies were rated in four categories: “high,” no critical weakness and no more than one noncritical weakness; “moderate,” no critical weakness and more than one noncritical weakness; “low,” one critical flaw with or without noncritical weaknesses; and “critically low,” more than one critical flaw with or without noncritical weaknesses. The evaluation results were presented through heat maps. Two reviewers (Yaning Zang and Xigui Lai) independently evaluated each study and rated the studies according to the AMSTAR-2 tool. Discrepancies in risk assessment were resolved by consensus and, if required, consultation with a third reviewer (Yi Zhu).

### 2.4. Data Extraction

Two reviewers (Yaning Zang and Dongfang Ding) independently extracted data using a predesigned table included: the author and year, study design included in this paper, search date of included study, study design and number of studies included in SRs, sample of SRs, flora sample, methods of microbiology assessment, participants type, diversity indices included *α* and *β* diversity indices of the microbiome, and gut microbiota's taxonomic composition at different levels, such as phylum, order, family, genus, and species.

### 2.5. Data Synthesis and Analysis

Studies included in this paper reported the comparison of gut microbiota between patients and controls, including alpha diversity, beta diversity, and the relative abundance of bacteria of different phylum, class, order, families, genus, and species. Evidence mapping was used to compare the different microbiota involved in the studies' pathologies. The evidence mapping displayed information in two dimensions: (1) The different colors show changes in the abundance of the flora in neurological or psychiatric disorders which included increase, decrease, significant difference, no difference, mixed, and unclear. It should be noted that when different studies show inconsistent changes in the microbiome, it was classified as mixed. (2) The different shapes in the cells indicate the strength of the evidence.

## 3. Results

### 3.1. Selected Studies

**Figure 1:**
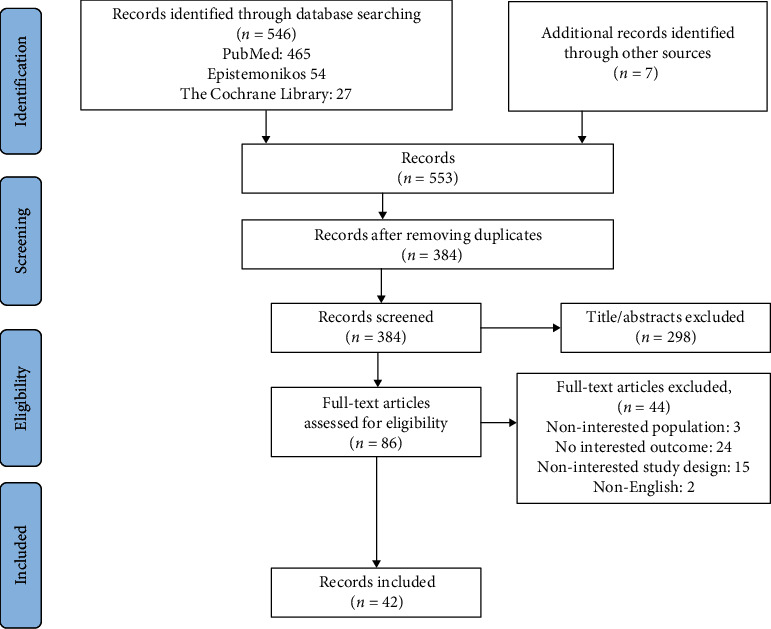
PRISMA flow diagram of the studies selection.

In total, 42 studies were included in this evidence mapping. A flow diagram of study selection is presented in Figure 1.

### 3.2. Methodological Quality of Included Studies

**Figure 2:**
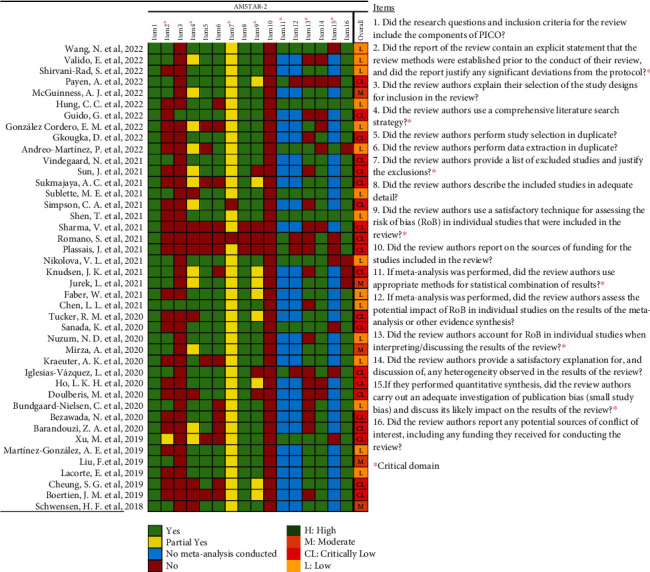
Methodological quality of the included studies.

According to the AMSTAR-2 criteria, 21 SRs [7, 17–36] scored “critically low,” 16 SR [13, 37–51] scored “low,” and 5 SR [36, 52–55] scored “moderate” (Figure 2). The most frequent drawbacks were as follows: no mentioning of the protocol in the systematic overview, no description of the rationale for the study designs included in the review, and no statement of funding for the included studies. The detailed assessments process is provided in Supplementary Material 2.

### 3.3. Characteristics of the Included Studies

The earliest articles included in this paper are from 2018. From 2018 forward, the number of studies in this field increased rapidly. Most of the primary studies were observational, including cohort studies, case-control studies, and case series. For the studies that used marker-gene analysis, 16S ribosomal RNA was the most amplified gene (Table 2).

The most studied neuropsychiatric disorder is autism spectrum disorder (ASD) with 11 studies [29, 30, 32, 34, 42, 46, 49–51, 53, 55] included in this paper. 3 SRs included Alzheimer's disease (AD) [31, 40, 41], 10 SRs included attention deficit hyperactivity disorder (ADHD) [17, 19, 22, 37, 39, 45, 46, 49, 51, 53], 1 SR included amyotrophic lateral sclerosis (ALS) [21], 2 SRs included anorexia nervosa (AN) [36, 45], 5 SRs included bipolar disorder (BD) [20, 43, 45, 46, 52], 1 SR included eating disorder (ED) [27], 2 SRs included generalized anxiety disorder (GAD) [45, 46], 9 SRs included major depressive disorder (MDD) [20, 23, 26, 28, 33, 45, 46, 52, 56], 3 SRs included multiple sclerosis (MS) [13, 25, 54], 1 SR included obsessive compulsive disorder (OCD) [45], 6 SRs included Parkinson's disease (PD) [7, 25, 27, 35, 44, 47], 2 SRs included posttraumatic stress disorder (PTSD) [45, 46], 2 SRs included spinal cord Injury (SCI) [13, 38], 5 SRs included schizophrenia [20, 45, 46, 48, 52], and 2 SRs included stroke [18, 24].

### 3.4. Specific Findings from the Evidence Mapping

**Figure 3:**
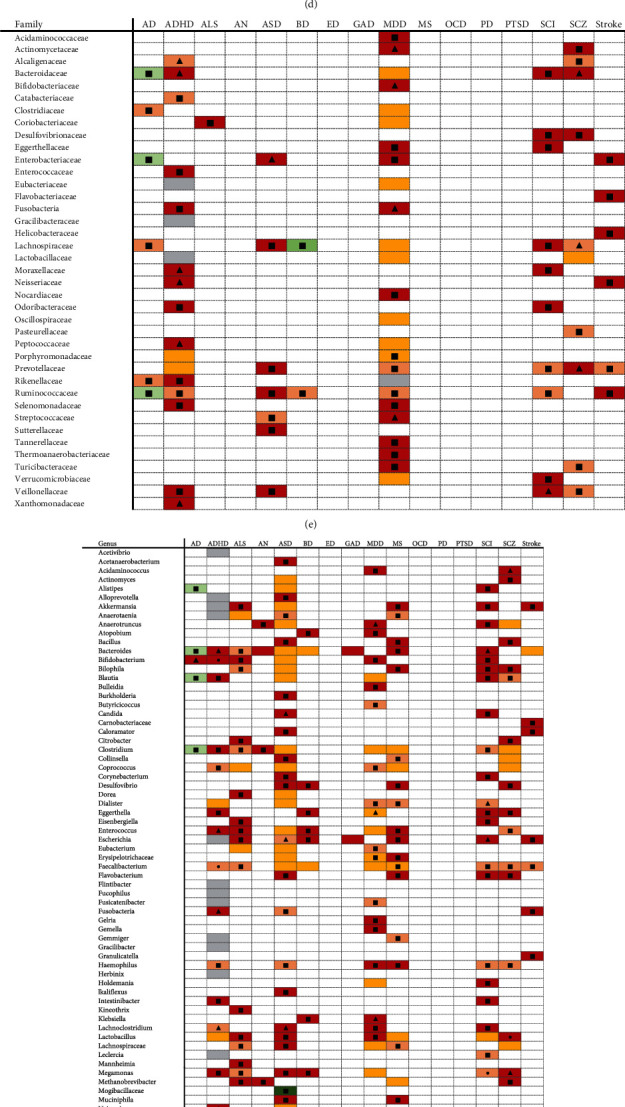
Evidence mapping of microbiome changes. Abbreviations: AD: Alzheimer's disease, ADHD: attention deficit hyperactivity disorder, ALS: amyotrophic lateral sclerosis, ASD: autism spectrum disorder, AN: anorexia nervosa, BD: bipolar disorder, ED: eating disorder; GAD: generalized anxiety disorder, MDD: major depressive disorder, MS: multiple sclerosis, OCD: obsessive compulsive disorder, PD: Parkinson's disease, PTSD: posttraumatic stress disorder, SCI: spinal cord injury, SCZ: schizophrenia. (a) Diversity. (b) Phylum level. (c) Class level. (d) Order level. (e) Family level. (f) Genus level. (g) Species level.

Figure 3 summarizes the outcomes of the included studies on microbiota profiles (alpha and beta diversity) and gut microbiota taxa. Studies included in this evidence mapping reported the comparison of gut microbiota between patients and controls, including alpha diversity, beta diversity, and the relative abundance of bacteria of different phylum, class, order, family, genus, and species.

A total of 15 diseases have been investigated for the potential association between gut microbiome alpha diversity and disease, with the Shannon index and Simpson index being the most widely studied. A total of 12 diseases were investigated for potential link between beta diversity and disease. The Bray-Curtis distance, weighted UniFrac distances, and unweighted UniFrac distances were the most widely examined. Regarding the microbiota assessment, it is the most classified the bacteria detected according to both phylum and genus, with a wide variety of bacteria being studied. Few studies included the level of species when classifying the bacteria detected. At the phylum level, 5 phyla were more identified: *Firmicutes*, *Bacteroidetes*, *Actinobacteria*, *Proteobacteria*, and *Verrucomicrobia*. At the genus level, 14 genera were more identified: *Prevotella*, *Coprococcus*, *Parabacteroides*, *Phascolarctobacterium*, *Escherichia Shigella*, *Alistipes*, *Sutteralla*, *Veillonella*, *Odoribacter*, *Faecalibacterium*, *Bacteroides*, *Bifidobacterium*, *Dialister*, and *Blautia*.

In particular, some diseases have been found to have specific flora changes. At the phylum level, *Coriobacteriaceae* was only observed in AN patients, and *Deferribacter*, *Lactobacillale*, and *Tropheryma* were only observed in SCZ patients. At the order level, *Alteromonadales*, *Bifidonbacteriales*, *Coriobacteriales*, *Cytophagales*, *Deltaproteobacteria*, *Eerysipelotrichales*, *Flavobacteriales*, *Pasteurellales*, and *Sphingobacteriales* were only observed in MDD patients, and *Desulfovibrio* was only observed in stroke patients. At the family level, *Acidaminococcaceae*, *Bifidobacteriaceae*, *Nocardiaceae*, *Tannerellaceae*, and *Thermoanaerobacteriaceae* were only observed in MDD patients, *Catabacteriaceae*, *Enterococcaceae*, and *Xanthomonadaceae* were only observed in ADHD patients, *Flavobacteriaceae* and *Helicobacteraceae* were only observed in stroke patients, *Pasteurellaceae* was only observed in SCZ patients, and *Sutterellaceae* was only observed in ASD patients. At the genus level, *Acetanaerobacterium*, *Burkholderia*, and *lkaliflexus* were only observed in ASD patients, *Kineothrix* was only observed in ALS patients, *Bulleidia*, *Butyricicoccus*, *Olsenella*, *Oxalobacter*, *Paraprevotella*, and *Parvimonas* were only observed in MDD patients, *Butyricicoccus* was only observed in stroke patients, and *Pseudomonas* was only observed in MS patients. At the species level, *Acidovorax* was only observed in stroke patients, *Bacteroides caccae*, *Bacteroides coprocola*, *Bacteroides ovatus*, *Bacteroides uniformis*, *Collinsella*, *Gemmiger*, *Lachnospiraceae*, *Lachnospiraceae*, *Lachnospiraceae*, *Streptococcus*, *Streptococcus*, *Uricibacter*, and *Veillonella parvula* were only observed in ADHD patients, *Bacteroidetes genera*, *Desulfovibrio*, *Devosia*, *Dialister invisus*, *Dialister invisus*, and *Dialister invisus* were only observed in ASD patients.

Although different studies draw many inconsistent conclusions, we found some overlaps between certain diseases when comparing the direction of association. At the phylum level, the most consistent change of ADHD, AN, and BD was the increase of *Actinobacteria*. The most consistent change of AD, ADHD, ALS, GAD, and MDD was the depletion of *Firmicutes*. At the genus level, the consistent change of ALS, MS, SCI, and stroke was the increase of *Akkermansia* and the increase of *Bacteroides* in ADHD, AN, GAD, MS, and SCI patients.

## 4. Discussion

### 4.1. Main Findings

In conclusion, our findings are as follows: first, studies on gut microbiomes among individuals with SCI, stroke, and AD are limited. The gastrointestinal symptoms (GI), such as diarrhea, constipation, and abdominal pain, inflammatory bowel disease, and irritable bowel syndrome (IBS) are often comorbid with SCI, stroke, AD, and so on. The simultaneous occurrence of neurological, psychiatric, and gastrointestinal diseases increases the risk of disease progression and poor outcomes, and the treatment of one disease can reverse the risk of another disease [5, 57]. There are still many gaps in whether modification of the gut microbiota can reduce the risk of these diseases or improve patient health.

Second, we found evidence of disease specificity, suggesting that these microbiota may be involved in the pathogenesis. And, identifying biotypes may provide opportunities to make targeted treatment strategies in clinic, including probiotic supplements, dietary changes, and even fecal microbial transplants (FMT). For example, *Lactobacillus rhamnosus* as a therapeutic supplement can reduce the risk of neuropsychiatric disorders in infants with autism spectrum disorders [58]. In a variety of animal models, *Lactobacillus* and *Bifidobacterium* can reduce the occurrence of anxiety and depression-related symptoms and positively affect memory, learning and cognition [59]. Nutritional deficiency due to inadequate intake or absorption is a recognized risk factor for neuropsychiatric diseases. For example, the content of folic acid and vitamin B12 in the blood of schizophrenic patients decreases and is related to the severity of symptoms [60]. Increase the intake of vegetables and fruits, restore the level of vitamin B in the body, and help to reduce and reverse some symptoms of neuropsychiatric diseases. Research found that a high-fat diet can significantly increase the deposition of amyloid protein and significantly increase the incidence of AD [61]. FMT infuses the fecal filtrate of healthy people into the intestines of patients with intestinal or neurological disorders to increase the number of beneficial bacteria and reduce the number of harmful bacteria in patients to maintain the steady state of gut microbiota [62, 63]. Neurologic dysfunction and autistic symptoms were significantly improved after FMT treatment in patients with MS and children with ASD [64].

Third, certain diseases have similar patterns of microbial changes. Specifically, we observed that ADHD, AN, and BD; AD, ADHD, ALS, GAD, and MDD; ALS, MS, SCI, and stroke; and ADHD, AN, GAD, MS, and SCI overlapped in the categories of changes in abundance, suggesting that these overlaps may be related to transdiagnostic of pathophysiology. There are several possible explanations for the mechanisms that drive the gut microbiota to affect different neurological and psychiatric disorders [6, 65, 66]: (1) Intestinal lymphocytes can feel the changes of gut microbiota, release endocrine or paracrine cytokines, and then act on the central nervous system; (2) intestinal peptide released by intestinal endocrine cells can stimulate sensory nerve endings, produce nerve impulses, and transmit them to the brain; and (3) microbial metabolites can act as neurotransmitters or their precursors on intestinal epithelial cells with endocrine or paracrine effects. Afferent stimuli relay through the brain stem and reach the visceral sensory higher center composed of amygdala and insula. For example, study [67] reported that the feces of PD patients had significantly reduced *Clostridium*, and the content of short chain fatty acids (SCFA) was significantly lower than that of healthy people, indicating that the intestinal ecological imbalance of PD patients was related to the decrease of SCFA level; then, the reduction of SCFA can promote *α*. The accumulation of synuclein in the intestinal nervous system leads to PD [68]. Although it is uncertain whether the change of intestinal flora is the cause or result of PD, gut microbiota can indeed lead to intestinal dysfunction and intestinal inflammation cascade through the interaction of intestinal epithelial barrier, immune system, and intestinal nervous system vagal pathway and then induce the loss of neuronal function [69].

Finally, it is worth noting that systematic reviews included in our study summarized differences in gut microbiota between the patients group and healthy group and draw a conflicting or even opposite conclusion, which may suggest the excess or dearth of a microbe may lead to deranged pathophysiology, so any microbe if not present in suitable amounts may be harmful [5]. Another reason for the nonsignificant difference may be that the observation of these studies is time limited. For example, after SCI, a new intestinal environment may allow new species to proliferate for a period of time, but in the end, these exceed the dominant species, resulting in a lack of changes in species abundance distribution.

### 4.2. Limitations and Further Direction

Due to the limitations of the included studies, we did not conduct analyses of sampling method, sampling time, sequencing, or analysis pipelines. Given that most findings were obtained from observational studies cannot infer causality or explain temporal changes in gut microbiota, so the possibility of reverse causality should be taken into account.

Another point that needs to be emphasized is that this article mainly analyzes the changes of single flora. Some studies have shown that the use of probiotics or probiotics may improve the symptoms of patients with neurological or mental disorders. In further research, we will analyze the effects of different probiotics, diets, and flora transplantation on patients to find the optimal combination of flora.

## 5. Conclusion

Analyzing the changes in the microbiome could be an essential source of knowledge for better understanding neurological or psychiatric disorders. Some diseases have specific flora changes, while others have consistent changes. Although the exact mechanism of action is unclear, regulating gut microbiota and maintaining physical stability and health by improving diet, supplementing special probiotics and probiotics, or FMT transplantation can open up new ideas for the treatment of neurological and mental diseases.

## Figures and Tables

**Table 1:** Inclusion and exclusion according to criteria.

Inclusion criteria	(i) Population patients with confirmed neurological or psychiatric disorders, such as AD, PD, ASD, and MMD (ii) Differences in gut microbiota diversity indices (alpha and beta diversity) and relative or absolute abundance of microbial taxa were reported between patients and healthy controls (iii) Systematic review or meta-analysis
Exclusion criteria	(i) Animal studies (ii) Healthy controls without neurological or psychiatric disorders (iii) Interventional studies. For example, the study is aimed at exploring the effects of probiotics or nutrition therapy (iv) Noninterested study design include conference papers, expert opinions, letters to the editor, or study protocol (v) Non-English article

**Table 2:** Characteristics of included systematic reviews.

Author and year	Study design	Search date	Number of studies included	Design and number of included studies	Sample	Microbiology assessment	Disease	Participants (*n*)
Wang, N. et al. 2022 [37]	Meta	August 24, 2021	8	Case-control	Fecal 8	16S rRNA gene sequencing 7 Shotgun metagenomics sequencing	ADHD	316
Valido, E. et al. 2022 [38]	SR	April 07, 2021	6	Case-control		16S rRNA 5 ASV clustering taxa assignment 1	SCI	246
Shirvani-Rad, S. et al. 2022 [39]	SR	March, 2021	8	Cohort 2 Case-control 6			ADHD	53886
Payen, A. et al. 2022 [17]	Meta	January 2021 to April 2021	5	Case-control	Fecal	16S rRNA 4 Shotgun metagenomic sequencing 1	ADHD	1134
McGuinness,A. J.et al. 2022 [52]	SR	December, 2021	44	Case-control			MDD	2086
							BD	1004
							SCZ	1827
Hung, C. C. et al. 2022 [40]	Meta	January 2000 to August 2021	11	Case-control		16S rRNA gene sequencing 11	AD	805
Guido, G. et al. 2022 [18]	SR	January, 2022	2	Case-control study	Fecal		Stroke	174
González Cordero, E. M. et al. 2022 [41]	SR	January 2016 to May 2020	8	Case-control 4 Longitudinal 4			AD	164182
Gkougka, D. et al. 2022 [19]	SR	December 31, 2020	11	Case-control studies 10			ADHD	54093
Andreo-Martínez, P. et al. 2022 [42]	Meta	January 27, 2020	18	Case-control			ASD	998
Vindegaard, N. et al. 2021 [20]	SR	January 17, 2019	17	Case-control 17			SCZ BD MDD	1364
Sun, J. et al. 2021 [21]	SR	February 2021	9	Case-control 8 randomized trial 1		Both 16S sequencing and shotgun metagenomic sequencing 2 16S-based approaches alone 6 Metagenomic sequencing 1	ALS	630
Sukmajaya, A. C. et al. 2021 [22]	SR	2017-2020	6			16S rRNA sequencing 4 DNA amplification 1 shotgun metagenomic sequencing 1	ADHD	407
Sublette, M. E. et al. 2021 [43]	SR	January 7, 2020	13	Case-control			BD	759
Simpson, C. A. et al. 2021 [23]	SR	March 2020	26	Case-control study			MDD	NA
Shen, T. et al. 2021 [44]	Meta	August 2020	15	Case-control studies	Fecal	Quantitative polymerase chain reaction (qPCR) 1 next-generation sequencing (NGS) technique.13	PD	1703
Sharma, V. et al. 2021 [24]	SR	January 1, 1990 to March 22, 2020	73	Case-control 8 Cohort 27 Clinical trial 1 Metagenomics (human) 11 Other (cross-sectional and experimental) 26			Stroke	NA
Romano, S. et al. 2021 [7]	Meta	March 29, 2020	10	Case-control			PD	1203
Plassais, J. et al. 2021 [25]	Meta	June 30, 2020	5	NA			MS	303
			7	NA			PD	1067
Nikolova, V. L.et al. 2021 [45]	Meta	January 27, 2021	59	Case-control studies		16S ribosomal RNA gene sequencing	MDD	930
							BD	465
							SCZ	699
							GAD	84
							AN	211
							PTSD	18
							OCD	59
							ADHD	19
Knudsen, J. K.et al. 2021 [26]	SR	November 13, 2020.	17	Case-control			MDD	1520
Jurek, L. et al. 2021 [53]	SR		31	Case-control	Stool samples 25 Urine samples 2 Intestinal biopsies 4	16S-targeted metagenomics 18 Real-time polymerase chain reaction (RT-PCR) 10 The FISH (fluorescent in situ hybridization) 1 extended-culture (culturomics) 10	ASD	3002
			3				ADHD	84
Faber, W. et al. 2021 [13]	SR		14	Case-control			MS	10
							SCI	4
Chen, L. L. et al. 2021 [46]	SR	February 13, 2020	69	Case-control		Marker-gene analysis methods 86% metagenome analysis 9%	ADHD	NA
							GAD	NA
							ASD	NA
							BD	NA
							ED	NA
							MDD	NA
							PTSD	NA
							SCZ	NA
Tucker, R. M.et al. 2020 [27]	SR	2000-2019	21	Case-control	UBT 3 stool antigen 1 serology 17		PD	48484
Sanada, K. et al. 2020 [28]	Meta	October 24, 2019	10	Observational		16S rRNA gene sequencing 9 Metaproteomics: phylogenetic analysis 1	MDD	701
Nuzum, N. D. et al. 2020 [47]	SR	May 27, 2018 to May 24, 2019	13	Case-control		Next-generation sequencing 11	PD	1587
Mirza, A. et al. 2020 [54]	SR	January 1, 2008 to august 24, 2019	10	Pilot study 3 Case-control 7	Stool 9 duodenal mucosa 1	16S rRNA 10	MS	582
Kraeuter, A. K. et al. 2020 [48]	SR	February 14, 2019	9	Case-control	Fecal sample		SCZ	594
Iglesias-Vázquez, L. et al. 2020 [29]	Meta	February, 2020	18	Case-control		Pyrosequencing 6 PCR 10 Culture 2	ASD	897
Ho, L. K. H. et al. 2020 [30]	SR	September 2017, August 2018, and April 2019	26	Case-control	Fecal 22 Gastric and duodenal fluids 1 Duodenal biopsy 1 Blood biopsy from colon1 Biopsy from ileum and cecum 1		ASD	1237
Doulberis, M.et al. 2020 [31]	SR	October 17, 2018	24	Randomized controlled trial 1 Prospective cohort study 9 Retrospective cohort study 4 Cross-sectional study 2 Case-control study 8			AD	10447
Bundgaard-Nielsen, C. et al. 2020 [49]	SR	July 22, 2019	24	Case-control		Metagenomic sequencing 2 sequencing of the 16S ribosomal ribonucleic acid (rRNA) gene 22	ASD	1323
							ADHD	270
Bezawada, N. et al. 2020 [32]	SR	1966 to July 2019	28	Case-control	Fecal samples 24 mucosal biopsies 4	16S r RNA 18 microbial analysis 4 quantitative real-time amplification of bacterial DNA (qPCR), 4 both qPCR and 16S rRNA sequencing techniques 1 fluorescent insitu hybridisation 1	ASD	1680
Barandouzi, Z. A. et al. 2020 [33]	SR	January 2000 to June 2019	9	Cross-sectional 8 Partially blinded observational study 1		16S rRNA 9	MDD	707
Xu, M. et al. 2019 [34]	Meta	July 2017	9	Cohort 1 NA: 8	Fecal 9	FISH (Cy3-labeled 16S rRNA probes) Pyrosequencing 5 QPCR (various bacterial primers) Culture (colony-forming units) 2	ASD	421
Martínez-González, A. E.et al. 2019 [50]	SR	Between 2012 and February 2019	16	Case-control	Stool	Sequencing of the 16S rRNA	ASD	508
Liu, F.et al. 2019 [55]	SR	March, 2018	16	Case-control	Fecal, 12 Rectal biopsy 1 Ileal and cecal biopsies 1	16S rRNA 12, quantitative real-time PCR 4	ASD	664
Lacorte, E. et al. 2019 [51]	SR	April, 2019	10	NA	Stool	NA	ADHD	114
							ASD	757
Cheung, S. G. et al. 2019 [56]	SR	February 28, 2018	6	Case-control	Stool		MDD	392
Boertien, J. M. et al. 2019 [35]	SR		16	Case-control		16S 13 qPCR of selected taxa 2 Shotgun meta genomics 1	PD	1804
Schwensen, H. F. et al. 2018 [36]	SR	August 27, 2017	10	Cross-sectional 6 longitudinal 2 case report 1 case series 1	Feces samples	16S reverse transcriptase-PCR 7 16S reverse transcriptase-PCR and 23S rRNA gene 1 NA 2	AN	731

Abbreviations: AD: Alzheimer's disease, ADHD: attention deficit hyperactivity disorder:, ALS: amyotrophic lateral sclerosis, ASD: autism spectrum disorder, AN: anorexia nervosa, BD: bipolar disorder, ED: eating disorder; GAD: generalized anxiety disorder, NA: not available, MDD: major depressive disorder, MS: multiple sclerosis, OCD: obsessive compulsive disorder, PD: Parkinson's disease, PTSD: posttraumatic stress disorder, SCI: spinal cord injury, SCZ: schizophrenia, SR: systematic review.

## Data Availability

All data supporting the conclusions of this study are included in the appendix.

## Supplementary Materials

Supplementary 1 Database search strategies.

Supplementary 2 AMSTAR-2 assessment.
