# Supplementary material for: The Role of Gut Microbiota in Various Neurological and Psychiatric Disorders—An Evidence Mapping Based on Quantified Evidence
Source: Mediators Inflamm. 2023 Feb 8;2023:5127157. doi: 10.1155/2023/5127157 (PMC9936509; doi:10.1155/2023/5127157)
Supplement: Supplementary 1 — Database search strategies. [file 5127157.f1.docx]

**Supplementary material 1: Search strategy**

| **Database** | **Terms** | **Results** |
| --- | --- | --- |
| PubMed | **#1: Nervous System Diseases** |  |
|  | ("Nervous System Diseases"[Mesh]) OR ((((((((((((((((Nervous System Diseases[Title/Abstract]) OR (Disease, Nervous System[Title/Abstract])) OR (Diseases, Nervous System[Title/Abstract])) OR (Nervous System Disease[Title/Abstract])) OR (Neurologic Disorders[Title/Abstract])) OR (Disorder, Neurologic[Title/Abstract])) OR (Disorders, Neurologic[Title/Abstract])) OR (Neurologic Disorder[Title/Abstract])) OR (Neurological Disorders[Title/Abstract])) OR (Disorder, Neurological[Title/Abstract])) OR (Disorders, Neurological[Title/Abstract])) OR (Neurological Disorder[Title/Abstract])) OR (Nervous System Disorders[Title/Abstract])) OR (Disorder, Nervous System[Title/Abstract])) OR (Disorders, Nervous System[Title/Abstract])) OR (Nervous System Disorder[Title/Abstract])) | 2751269 |
|  | **#2： Neurocognitive Disorders** |  |
|  | ("Neurocognitive Disorders"[Mesh]) OR (((((((((((((((((Neurocognitive Disorders[Title/Abstract]) OR (Disorder, Neurocognitive[Title/Abstract])) OR (Disorders, Neurocognitive[Title/Abstract])) OR (Neurocognitive Disorder[Title/Abstract])) OR (Organic Brain Syndrome, Nonpsychotic[Title/Abstract])) OR (Nonpsychotic Organic Brain Syndrome[Title/Abstract])) OR (Organic Mental Disorders, Psychotic[Title/Abstract])) OR (Psychoses, Traumatic[Title/Abstract])) OR (Traumatic Psychoses[Title/Abstract])) OR (Delirium, Dementia, Amnestic, Cognitive Disorders[Title/Abstract])) OR (Mental Disorders, Organic[Title/Abstract])) OR (Disorders, Organic Mental[Title/Abstract])) OR (Mental Disorder, Organic[Title/Abstract])) OR (Organic Mental Disorder[Title/Abstract])) OR (Organic Mental Disorders[Title/Abstract])) OR (Kandinsky Syndrome[Title/Abstract])) OR (Clerambault Syndrome[Title/Abstract])) | 292036 |
|  | **#3： Central Nervous System** |  |
|  | ("Central Nervous System"[Mesh]) OR (((((((((Central Nervous System[Title/Abstract]) OR (Central Nervous Systems[Title/Abstract])) OR (Nervous System, Central[Title/Abstract])) OR (Nervous Systems, Central[Title/Abstract])) OR (Systems, Central Nervous[Title/Abstract])) OR (Cerebrospinal Axis[Title/Abstract])) OR (Axi, Cerebrospinal[Title/Abstract])) OR (Axis, Cerebrospinal[Title/Abstract])) OR (Cerebrospinal Axi[Title/Abstract])) | 1530932 |
|  | **#4: Mental Disorders** |  |
|  | ("Mental Disorders"[Mesh]) OR ((((((((((((((((((Mental Disorders[Title/Abstract]) OR (Mental Disorder[Title/Abstract])) OR (Psychiatric Illness[Title/Abstract])) OR (Psychiatric Illnesses[Title/Abstract])) OR (Psychiatric Diseases[Title/Abstract])) OR (Psychiatric Disease[Title/Abstract])) OR (Mental Illness[Title/Abstract])) OR (Illness, Mental[Title/Abstract])) OR (Mental Illnesses[Title/Abstract])) OR (Psychiatric Disorders[Title/Abstract])) OR (Psychiatric Disorder[Title/Abstract])) OR (Behavior Disorders[Title/Abstract])) OR (Diagnosis, Psychiatric[Title/Abstract])) OR (Psychiatric Diagnosis[Title/Abstract])) OR (Mental Disorders, Severe[Title/Abstract])) OR (Mental Disorder, Severe[Title/Abstract])) OR (Severe Mental Disorder[Title/Abstract])) OR (Severe Mental Disorders[Title/Abstract])) | 1410962 |
|  | **#5: Brain Injuries** |  |
|  | ("Brain Injuries"[Mesh]) OR ((((((((((((((((((((Brain Injuries[Title/Abstract]) OR (Injuries, Brain[Title/Abstract])) OR (Brain Injury[Title/Abstract])) OR (Injury, Brain[Title/Abstract])) OR (Injuries, Acute Brain[Title/Abstract])) OR (Acute Brain Injuries[Title/Abstract])) OR (Acute Brain Injury[Title/Abstract])) OR (Brain Injury, Acute[Title/Abstract])) OR (Injury, Acute Brain[Title/Abstract])) OR (Brain Injuries, Acute[Title/Abstract])) OR (Brain Lacerations[Title/Abstract])) OR (Brain Laceration[Title/Abstract])) OR (Laceration, Brain[Title/Abstract])) OR (Lacerations, Brain[Title/Abstract])) OR (Brain Injuries, Focal[Title/Abstract])) OR (Brain Injury, Focal[Title/Abstract])) OR (Focal Brain Injury[Title/Abstract])) OR (Injuries, Focal Brain[Title/Abstract])) OR (Injury, Focal Brain[Title/Abstract])) OR (Focal Brain Injuries[Title/Abstract])) | 112865 |
|  | **#6: Parkinson Disease** |  |
|  | ("Parkinson Disease"[Mesh]) OR (((((((((((((Parkinson disease[Title/Abstract]) OR (Idiopathic Parkinson's Disease[Title/Abstract])) OR (Lewy Body Parkinson's Disease[Title/Abstract])) OR (Parkinson's Disease, Idiopathic[Title/Abstract])) OR (Parkinson's Disease, Lewy Body[Title/Abstract])) OR (Parkinson Disease, Idiopathic[Title/Abstract])) OR (Parkinson's Disease[Title/Abstract])) OR (Idiopathic Parkinson Disease[Title/Abstract])) OR (Lewy Body Parkinson Disease[Title/Abstract])) OR (Primary Parkinsonism[Title/Abstract])) OR (Parkinsonism, Primary[Title/Abstract])) OR (Paralysis Agitans[Title/Abstract])) OR (PD[Title/Abstract])) | 233677 |
|  | **#7: Multiple Sclerosis** |  |
|  | ("Multiple Sclerosis"[Mesh]) OR ((((((Multiple Sclerosis[Title/Abstract]) OR (Sclerosis, Multiple[Title/Abstract])) OR (Sclerosis, Disseminated[Title/Abstract])) OR (Disseminated Sclerosis[Title/Abstract])) OR (MS (Multiple Sclerosis[Title/Abstract]))) OR (Multiple Sclerosis, Acute Fulminating[Title/Abstract])) | 92934 |
|  | **#8: Alzheimer's disease** |  |
|  | ("Alzheimer Disease"[Mesh]) OR ((((((((((((((((((((((((((((((((((((Alzheimer Disease[Title/Abstract]) OR (Alzheimer Dementia[Title/Abstract])) OR (Alzheimer Dementias[Title/Abstract])) OR (Dementia, Alzheimer[Title/Abstract])) OR (Alzheimer's Disease[Title/Abstract])) OR (Dementia, Senile[Title/Abstract])) OR (Senile Dementia[Title/Abstract])) OR (Dementia, Alzheimer Type[Title/Abstract])) OR (Alzheimer Type Dementia[Title/Abstract])) OR (Alzheimer-Type Dementia (ATD[Title/Abstract]))) OR (Alzheimer Type Dementia (ATD[Title/Abstract]))) OR (Dementia, Alzheimer-Type (ATD[Title/Abstract]))) OR (Alzheimer Type Senile Dementia[Title/Abstract])) OR (Primary Senile Degenerative Dementia[Title/Abstract])) OR (Dementia, Primary Senile Degenerative[Title/Abstract])) OR (Alzheimer Sclerosis[Title/Abstract])) OR (Sclerosis, Alzheimer[Title/Abstract])) OR (Alzheimer Syndrome[Title/Abstract])) OR (Alzheimer's Diseases[Title/Abstract])) OR (Alzheimer Diseases[Title/Abstract])) OR (Alzheimers Diseases[Title/Abstract])) OR (Senile Dementia, Alzheimer Type[Title/Abstract])) OR (Acute Confusional Senile Dementia[Title/Abstract])) OR (Senile Dementia, Acute Confusional[Title/Abstract])) OR (Dementia, Presenile[Title/Abstract])) OR (Presenile Dementia[Title/Abstract])) OR (Alzheimer Disease, Late Onset[Title/Abstract])) OR (Late Onset Alzheimer Disease[Title/Abstract])) OR (Alzheimer's Disease, Focal Onset[Title/Abstract])) OR (Focal Onset Alzheimer's Disease[Title/Abstract])) OR (Familial Alzheimer Disease (FAD[Title/Abstract]))) OR (Alzheimer Disease, Familial (FAD[Title/Abstract]))) OR (Familial Alzheimer Diseases (FAD[Title/Abstract]))) OR (Alzheimer Disease, Early Onset[Title/Abstract])) OR (Early Onset Alzheimer Disease[Title/Abstract])) OR (Presenile Alzheimer Dementia[Title/Abstract])) | 175711 |
|  | **#9: Brain Ischemia** |  |
|  | ("Brain Ischemia"[Mesh]) OR ((((((((((Brain Ischemia[Title/Abstract]) OR (Brain Ischemias[Title/Abstract])) OR (Ischemia, Brain[Title/Abstract])) OR (Ischemic Encephalopathy[Title/Abstract])) OR (Encephalopathy, Ischemic[Title/Abstract])) OR (Ischemic Encephalopathies[Title/Abstract])) OR (Cerebral Ischemia[Title/Abstract])) OR (Cerebral Ischemias[Title/Abstract])) OR (Ischemias, Cerebral[Title/Abstract])) OR (Ischemia, Cerebral[Title/Abstract])) | 140058 |
|  | **#10: Intracranial Hemorrhages** |  |
|  | ("Intracranial Hemorrhages"[Mesh]) OR ((((((((((((Intracranial Hemorrhages[Title/Abstract]) OR (Hemorrhages, Intracranial[Title/Abstract])) OR (Intracranial Hemorrhage[Title/Abstract])) OR (Hemorrhage, Intracranial[Title/Abstract])) OR (Posterior Fossa Hemorrhage[Title/Abstract])) OR (Hemorrhage, Posterior Fossa[Title/Abstract])) OR (Hemorrhages, Posterior Fossa[Title/Abstract])) OR (Posterior Fossa Hemorrhages[Title/Abstract])) OR (Brain Hemorrhage[Title/Abstract])) OR (Brain Hemorrhages[Title/Abstract])) OR (Hemorrhage, Brain[Title/Abstract])) OR (Hemorrhages, Brain[Title/Abstract])) | 85877 |
|  | **#11: Hepatic Encephalopathy** |  |
|  | ("Hepatic Encephalopathy"[Mesh]) OR (((((((((((((((((((((((((((Hepatic encephalopathy[Title/Abstract]) OR (Encephalopathies, Hepatic[Title/Abstract])) OR (Hepatic Encephalopathies[Title/Abstract])) OR (Encephalopathy, Hepatic[Title/Abstract])) OR (Portal-Systemic Encephalopathy[Title/Abstract])) OR (Portal Systemic Encephalopathy[Title/Abstract])) OR (Encephalopathy, Portal-Systemic[Title/Abstract])) OR (Encephalopathies, Portal-Systemic[Title/Abstract])) OR (Encephalopathy, Portal Systemic[Title/Abstract])) OR (Portal-Systemic Encephalopathies[Title/Abstract])) OR (Encephalopathy, Portosystemic[Title/Abstract])) OR (Hepatocerebral Encephalopathy[Title/Abstract])) OR (Portosystemic Encephalopathy[Title/Abstract])) OR (Encephalopathies, Portosystemic[Title/Abstract])) OR (Portosystemic Encephalopathies[Title/Abstract])) OR (Encephalopathy, Hepatocerebral[Title/Abstract])) OR (Encephalopathies, Hepatocerebral[Title/Abstract])) OR (Hepatocerebral Encephalopathies[Title/Abstract])) OR (Hepatic Coma[Title/Abstract])) OR (Coma, Hepatic[Title/Abstract])) OR (Comas, Hepatic[Title/Abstract])) OR (Hepatic Comas[Title/Abstract])) OR (Hepatic Stupor[Title/Abstract])) OR (Hepatic Stupors[Title/Abstract])) OR (Stupor, Hepatic[Title/Abstract])) OR (Stupors, Hepatic[Title/Abstract])) OR (Fulminant Hepatic Failure with Cerebral Edema[Title/Abstract])) | 17228 |
|  | **#12: Stroke** |  |
|  | ("Stroke"[Mesh]) OR (((((((((((((((((((((((((((((Stroke[Title/Abstract]) OR (Strokes[Title/Abstract])) OR (Cerebrovascular Accident[Title/Abstract])) OR (Cerebrovascular Accidents[Title/Abstract])) OR (CVA (Cerebrovascular Accident[Title/Abstract]))) OR (CVAs (Cerebrovascular Accident[Title/Abstract]))) OR (Cerebrovascular Apoplexy[Title/Abstract])) OR (Apoplexy, Cerebrovascular[Title/Abstract])) OR (Vascular Accident, Brain[Title/Abstract])) OR (Brain Vascular Accident[Title/Abstract])) OR (Brain Vascular Accidents[Title/Abstract])) OR (Vascular Accidents, Brain[Title/Abstract])) OR (Cerebrovascular Stroke[Title/Abstract])) OR (Cerebrovascular Strokes[Title/Abstract])) OR (Stroke, Cerebrovascular[Title/Abstract])) OR (Strokes, Cerebrovascular[Title/Abstract])) OR (Apoplexy[Title/Abstract])) OR (Cerebral Stroke[Title/Abstract])) OR (Cerebral Strokes[Title/Abstract])) OR (Stroke, Cerebral[Title/Abstract])) OR (Strokes, Cerebral[Title/Abstract])) OR (Stroke, Acute[Title/Abstract])) OR (Acute Stroke[Title/Abstract])) OR (Acute Strokes[Title/Abstract])) OR (Strokes, Acute[Title/Abstract])) OR (Cerebrovascular Accident, Acute[Title/Abstract])) OR (Acute Cerebrovascular Accident[Title/Abstract])) OR (Acute Cerebrovascular Accidents[Title/Abstract])) OR (Cerebrovascular Accidents, Acute[Title/Abstract])) | 335452 |
|  | **#13 Spinal Cord Injuries** |  |
|  | ("Spinal Cord Injuries"[Mesh]) OR (((((((((((((((((((((((((((((((((((((((Spinal Cord Injuries[Title/Abstract]) OR (Spinal Cord Trauma[Title/Abstract])) OR (Cord Trauma, Spinal[Title/Abstract])) OR (Cord Traumas, Spinal[Title/Abstract])) OR (Spinal Cord Traumas[Title/Abstract])) OR (Trauma, Spinal Cord[Title/Abstract])) OR (Traumas, Spinal Cord[Title/Abstract])) OR (Myelopathy, Traumatic[Title/Abstract])) OR (Myelopathies, Traumatic[Title/Abstract])) OR (Traumatic Myelopathies[Title/Abstract])) OR (Traumatic Myelopathy[Title/Abstract])) OR (Injuries, Spinal Cord[Title/Abstract])) OR (Cord Injuries, Spinal[Title/Abstract])) OR (Cord Injury, Spinal[Title/Abstract])) OR (Injury, Spinal Cord[Title/Abstract])) OR (Spinal Cord Injury[Title/Abstract])) OR (Spinal Cord Transection[Title/Abstract])) OR (Cord Transection, Spinal[Title/Abstract])) OR (Cord Transections, Spinal[Title/Abstract])) OR (Spinal Cord Transections[Title/Abstract])) OR (Transection, Spinal Cord[Title/Abstract])) OR (Transections, Spinal Cord[Title/Abstract])) OR (Spinal Cord Laceration[Title/Abstract])) OR (Cord Laceration, Spinal[Title/Abstract])) OR (Cord Lacerations, Spinal[Title/Abstract])) OR (Laceration, Spinal Cord[Title/Abstract])) OR (Lacerations, Spinal Cord[Title/Abstract])) OR (Spinal Cord Lacerations[Title/Abstract])) OR (Post-Traumatic Myelopathy[Title/Abstract])) OR (Myelopathies, Post-Traumatic[Title/Abstract])) OR (Myelopathy, Post-Traumatic[Title/Abstract])) OR (Post Traumatic Myelopathy[Title/Abstract])) OR (Post-Traumatic Myelopathies[Title/Abstract])) OR (Spinal Cord Contusion[Title/Abstract])) OR (Contusion, Spinal Cord[Title/Abstract])) OR (Contusions, Spinal Cord[Title/Abstract])) OR (Cord Contusion, Spinal[Title/Abstract])) OR (Cord Contusions, Spinal[Title/Abstract])) OR (Spinal Cord Contusions[Title/Abstract]) | 73972 |
|  | **#14 Traumatic Brain Injury** |  |
|  | ("Brain Injuries, Traumatic"[Mesh]) OR ((((((((((((((((Brain Injuries, Traumatic[Title/Abstract]) OR (Brain Injury, Traumatic[Title/Abstract])) OR (Traumatic Brain Injuries[Title/Abstract])) OR (Trauma, Brain[Title/Abstract])) OR (Brain Trauma[Title/Abstract])) OR (Brain Traumas[Title/Abstract])) OR (Traumas, Brain[Title/Abstract])) OR (TBI (Traumatic Brain Injury[Title/Abstract]))) OR (Encephalopathy, Traumatic[Title/Abstract])) OR (Encephalopathies, Traumatic[Title/Abstract])) OR (Traumatic Encephalopathies[Title/Abstract])) OR (Injury, Brain, Traumatic[Title/Abstract])) OR (Traumatic Encephalopathy[Title/Abstract])) OR (TBIs (Traumatic Brain Injuries[Title/Abstract]))) OR (TBI (Traumatic Brain Injuries[Title/Abstract]))) OR (Traumatic Brain Injury[Title/Abstract])) | 65394 |
|  | **#15 Amyotrophic Lateral Sclerosis** |  |
|  | ("Amyotrophic Lateral Sclerosis"[Mesh]) OR (((((((((((((((((((((((Amyotrophic Lateral Sclerosis[Title/Abstract]) OR (Sclerosis, Amyotrophic Lateral[Title/Abstract])) OR (Gehrig's Disease[Title/Abstract])) OR (Gehrig Disease[Title/Abstract])) OR (Gehrigs Disease[Title/Abstract])) OR (Charcot Disease[Title/Abstract])) OR (Motor Neuron Disease, Amyotrophic Lateral Sclerosis[Title/Abstract])) OR (Lou Gehrig's Disease[Title/Abstract])) OR (Lou-Gehrigs Disease[Title/Abstract])) OR (Disease, Lou-Gehrigs[Title/Abstract])) OR (ALS - Amyotrophic Lateral Sclerosis[Title/Abstract])) OR (ALS Amyotrophic Lateral Sclerosis[Title/Abstract])) OR (Lou Gehrig Disease[Title/Abstract])) OR (Amyotrophic Lateral Sclerosis, Guam Form[Title/Abstract])) OR (Amyotrophic Lateral Sclerosis-Parkinsonism-Dementia Complex 1[Title/Abstract])) OR (Amyotrophic Lateral Sclerosis Parkinsonism Dementia Complex 1[Title/Abstract])) OR (Guam Form of Amyotrophic Lateral Sclerosis[Title/Abstract])) OR (Guam Disease[Title/Abstract])) OR (Disease, Guam[Title/Abstract])) OR (Amyotrophic Lateral Sclerosis, Parkinsonism-Dementia Complex of Guam[Title/Abstract])) OR (Amyotrophic Lateral Sclerosis, Parkinsonism Dementia Complex of Guam[Title/Abstract])) OR (Amyotrophic Lateral Sclerosis With Dementia[Title/Abstract])) OR (Dementia With Amyotrophic Lateral Sclerosis[Title/Abstract])) | 3599929 |
|  | **#16 Epilepsy** |  |
|  | ("Epilepsy"[Mesh]) OR ((((((((((((Epilepsy[Title/Abstract]) OR (Epilepsies[Title/Abstract])) OR (Seizure Disorder[Title/Abstract])) OR (Seizure Disorders[Title/Abstract])) OR (Awakening Epilepsy[Title/Abstract])) OR (Epilepsy, Awakening[Title/Abstract])) OR (Epilepsy, Cryptogenic[Title/Abstract])) OR (Cryptogenic Epilepsies[Title/Abstract])) OR (Cryptogenic Epilepsy[Title/Abstract])) OR (Epilepsies, Cryptogenic[Title/Abstract])) OR (Aura[Title/Abstract])) OR (Auras[Title/Abstract])) | 168543 |
|  | **#17 Autism Spectrum Disorder** |  |
|  | ("Autism Spectrum Disorder"[Mesh]) OR (((((Autism Spectrum Disorder[Title/Abstract]) OR (Autism Spectrum Disorders[Title/Abstract])) OR (Autistic Spectrum Disorder[Title/Abstract])) OR (Autistic Spectrum Disorders[Title/Abstract])) OR (Disorder, Autistic Spectrum[Title/Abstract])) | 51697 |
|  | **#18 Attention Deficit Disorder with Hyperactivity** |  |
|  | ("Attention Deficit Disorder with Hyperactivity"[Mesh]) OR (((((((((((((((((((((((Attention Deficit Disorder with Hyperactivity[Title/Abstract]) OR (Attention Deficit Disorders with Hyperactivity[Title/Abstract])) OR (ADHD[Title/Abstract])) OR (Attention Deficit Hyperactivity Disorder[Title/Abstract])) OR (Hyperkinetic Syndrome[Title/Abstract])) OR (Syndromes, Hyperkinetic[Title/Abstract])) OR (Attention Deficit-Hyperactivity Disorder[Title/Abstract])) OR (Attention Deficit-Hyperactivity Disorders[Title/Abstract])) OR (Deficit-Hyperactivity Disorder, Attention[Title/Abstract])) OR (Deficit-Hyperactivity Disorders, Attention[Title/Abstract])) OR (Disorder, Attention Deficit-Hyperactivity[Title/Abstract])) OR (Disorders, Attention Deficit-Hyperactivity[Title/Abstract])) OR (ADDH[Title/Abstract])) OR (Attention Deficit Hyperactivity Disorders[Title/Abstract])) OR (Attention Deficit Disorder[Title/Abstract])) OR (Attention Deficit Disorders[Title/Abstract])) OR (Deficit Disorder, Attention[Title/Abstract])) OR (Deficit Disorders, Attention[Title/Abstract])) OR (Disorder, Attention Deficit[Title/Abstract])) OR (Disorders, Attention Deficit[Title/Abstract])) OR (Brain Dysfunction, Minimal[Title/Abstract])) OR (Dysfunction, Minimal Brain[Title/Abstract])) OR (Minimal Brain Dysfunction[Title/Abstract])) | 45918 |
|  | **#19 Hyperkinesis** |  |
|  | ("Hyperkinesis"[Mesh]) OR ((((((((((((Hyperkinesis[Title/Abstract]) OR (Hyperkinesia[Title/Abstract])) OR (Hyperkinetic Movements[Title/Abstract])) OR (Hyperkinetic Movement[Title/Abstract])) OR (Movement, Hyperkinetic[Title/Abstract])) OR (Movements, Hyperkinetic[Title/Abstract])) OR (Hyperactivity, Motor[Title/Abstract])) OR (Motor Hyperactivity[Title/Abstract])) OR (Hyperkinesia, Generalized[Title/Abstract])) OR (Generalized Hyperkinesia[Title/Abstract])) OR (Generalized Hyperkinesias[Title/Abstract])) OR (Hyperkinesias, Generalized[Title/Abstract])) | 6662 |
|  | **# 20 schizophrenia** |  |
|  | ("Schizophrenia"[Mesh]) OR (((((((schizophrenia[Title/Abstract]) OR (Schizophrenias[Title/Abstract])) OR (Schizophrenic Disorders[Title/Abstract])) OR (Disorder, Schizophrenic[Title/Abstract])) OR (Disorders, Schizophrenic[Title/Abstract])) OR (Schizophrenic Disorder[Title/Abstract])) OR (Dementia Praecox[Title/Abstract])) | 153352 |
|  | **# 21 bipolar disorder** |  |
|  | ("Bipolar Disorder"[Mesh]) OR (((((((((((((((((((((((((Bipolar Disorder[Title/Abstract]) OR (Bipolar Disorders[Title/Abstract])) OR (Disorder, Bipolar[Title/Abstract])) OR (Affective Psychosis, Bipolar[Title/Abstract])) OR (Bipolar Affective Psychosis[Title/Abstract])) OR (Psychoses, Bipolar Affective[Title/Abstract])) OR (Psychosis, Bipolar Affective[Title/Abstract])) OR (Manic-Depressive Psychosis[Title/Abstract])) OR (Manic Depressive Psychosis[Title/Abstract])) OR (Psychosis, Manic-Depressive[Title/Abstract])) OR (Psychosis, Manic Depressive[Title/Abstract])) OR (Bipolar Mood Disorder[Title/Abstract])) OR (Bipolar Mood Disorders[Title/Abstract])) OR (Disorder, Bipolar Mood[Title/Abstract])) OR (Mood Disorder, Bipolar[Title/Abstract])) OR (Psychoses, Manic-Depressive[Title/Abstract])) OR (Psychoses, Manic Depressive[Title/Abstract])) OR (Depression, Bipolar[Title/Abstract])) OR (Bipolar Depression[Title/Abstract])) OR (Manic Depression[Title/Abstract])) OR (Depression, Manic[Title/Abstract])) OR (Depressions, Manic[Title/Abstract])) OR (Manic Disorder[Title/Abstract])) OR (Disorder, Manic[Title/Abstract])) OR (Manic Disorders[Title/Abstract])) | 55874 |
|  | **# 22 Cognitive Dysfunction** |  |
|  | ("Cognitive Dysfunction"[Mesh]) OR ((((((((((((((((((((((((((((Cognitive Dysfunction[Title/Abstract]) OR (Cognitive Dysfunctions[Title/Abstract])) OR (Dysfunction, Cognitive[Title/Abstract])) OR (Dysfunctions, Cognitive[Title/Abstract])) OR (Cognitive Impairments[Title/Abstract])) OR (Cognitive Impairment[Title/Abstract])) OR (Impairment, Cognitive[Title/Abstract])) OR (Impairments, Cognitive[Title/Abstract])) OR (Mild Cognitive Impairment[Title/Abstract])) OR (Cognitive Impairment, Mild[Title/Abstract])) OR (Cognitive Impairments, Mild[Title/Abstract])) OR (Impairment, Mild Cognitive[Title/Abstract])) OR (Impairments, Mild Cognitive[Title/Abstract])) OR (Mild Cognitive Impairments[Title/Abstract])) OR (Mild Neurocognitive Disorder[Title/Abstract])) OR (Disorder, Mild Neurocognitive[Title/Abstract])) OR (Disorders, Mild Neurocognitive[Title/Abstract])) OR (Mild Neurocognitive Disorders[Title/Abstract])) OR (Neurocognitive Disorder, Mild[Title/Abstract])) OR (Neurocognitive Disorders, Mild[Title/Abstract])) OR (Cognitive Decline[Title/Abstract])) OR (Cognitive Declines[Title/Abstract])) OR (Decline, Cognitive[Title/Abstract])) OR (Declines, Cognitive[Title/Abstract])) OR (Mental Deterioration[Title/Abstract])) OR (Deterioration, Mental[Title/Abstract])) OR (Deteriorations, Mental[Title/Abstract])) OR (Mental Deteriorations[Title/Abstract])) | 134595 |
|  | **# 23 Alexithymia** |  |
|  | ("Affective Symptoms"[Mesh]) OR ((((((((((Affective Symptoms[Title/Abstract]) OR (Affective Symptom[Title/Abstract])) OR (Symptom, Affective[Title/Abstract])) OR (Symptoms, Affective[Title/Abstract])) OR (Alexithymia[Title/Abstract])) OR (Alexithymias[Title/Abstract])) OR (Emotional Disturbances[Title/Abstract])) OR (Disturbance, Emotional[Title/Abstract])) OR (Disturbances, Emotional[Title/Abstract])) OR (Emotional Disturbance[Title/Abstract])) | 18443 |
|  | **# 24 Depressive Disorder** |  |
|  | ("Depressive Disorder"[Mesh]) OR ((((((((((((((((((((((((((Depressive Disorder[Title/Abstract]) OR (Depressive Disorders[Title/Abstract])) OR (Disorder, Depressive[Title/Abstract])) OR (Disorders, Depressive[Title/Abstract])) OR (Neurosis, Depressive[Title/Abstract])) OR (Depressive Neuroses[Title/Abstract])) OR (Depressive Neurosis[Title/Abstract])) OR (Neuroses, Depressive[Title/Abstract])) OR (Depression, Endogenous[Title/Abstract])) OR (Depressions, Endogenous[Title/Abstract])) OR (Endogenous Depression[Title/Abstract])) OR (Endogenous Depressions[Title/Abstract])) OR (Depressive Syndrome[Title/Abstract])) OR (Depressive Syndromes[Title/Abstract])) OR (Syndrome, Depressive[Title/Abstract])) OR (Syndromes, Depressive[Title/Abstract])) OR (Depression, Neurotic[Title/Abstract])) OR (Depressions, Neurotic[Title/Abstract])) OR (Neurotic Depression[Title/Abstract])) OR (Neurotic Depressions[Title/Abstract])) OR (Melancholia[Title/Abstract])) OR (Melancholias[Title/Abstract])) OR (Unipolar Depression[Title/Abstract])) OR (Depression, Unipolar[Title/Abstract])) OR (Depressions, Unipolar[Title/Abstract])) OR (Unipolar Depressions[Title/Abstract])) | 137236 |
|  | **# 25 Dementia** |  |
|  | ("Dementia"[Mesh]) OR (((((((((((((Dementia[Title/Abstract]) OR (Dementias[Title/Abstract])) OR (Amentia[Title/Abstract])) OR (Amentias[Title/Abstract])) OR (Senile Paranoid Dementia[Title/Abstract])) OR (Dementias, Senile Paranoid[Title/Abstract])) OR (Paranoid Dementia, Senile[Title/Abstract])) OR (Paranoid Dementias, Senile[Title/Abstract])) OR (Senile Paranoid Dementias[Title/Abstract])) OR (Familial Dementia[Title/Abstract])) OR (Dementia, Familial[Title/Abstract])) OR (Dementias, Familial[Title/Abstract])) OR (Familial Dementias[Title/Abstract])) | 238601 |
|  | # **26 Anxiety Disorders** |  |
|  | ("Anxiety Disorders"[Mesh]) OR ((((((((((((Anxiety Disorders[Title/Abstract]) OR (Anxiety Disorder[Title/Abstract])) OR (Disorder, Anxiety[Title/Abstract])) OR (Disorders, Anxiety[Title/Abstract])) OR (Neuroses, Anxiety[Title/Abstract])) OR (Anxiety Neuroses[Title/Abstract])) OR (Anxiety States, Neurotic[Title/Abstract])) OR (Anxiety State, Neurotic[Title/Abstract])) OR (Neurotic Anxiety State[Title/Abstract])) OR (Neurotic Anxiety States[Title/Abstract])) OR (State, Neurotic Anxiety[Title/Abstract])) OR (States, Neurotic Anxiety[Title/Abstract])) | 104824 |
|  | **# 27 : #1 OR #3 OR #4 OR #5 OR #6 OR #7 OR #8 OR #9 OR #10 OR #11 OR #12 OR #13 OR #14 OR #15 OR #16 OR #17 OR #18 OR #19 OR #20 OR #21 OR #22 OR #23 OR #24 OR #25 OR #26** | 7780364 |
|  | **#28 Gut-Brain Axis** |  |
|  | ("Brain-Gut Axis"[Mesh]) OR ((((((((((((((((((((((((((Gut-Brain Axis[Title/Abstract]) OR (Axis, Brain-Gut[Title/Abstract])) OR (Brain Gut Axis[Title/Abstract])) OR (Gut[Title/Abstract] AND Brain Axis[Title/Abstract])) OR (Gut-Brain Axis[Title/Abstract])) OR (Axis, Gut-Brain[Title/Abstract])) OR (Gut Brain Axis[Title/Abstract])) OR (Brain[Title/Abstract] AND Gut Axis[Title/Abstract])) OR (Microbiota-Gut-Brain Axis[Title/Abstract])) OR (Axis, Microbiota-Gut-Brain[Title/Abstract])) OR (Microbiota Gut Brain Axis[Title/Abstract])) OR (Brain-Gut-Microbiome Axis[Title/Abstract])) OR (Axis, Brain-Gut-Microbiome[Title/Abstract])) OR (Brain Gut Microbiome Axis[Title/Abstract])) OR (Microbiome-Gut-Brain Axis[Title/Abstract])) OR (Axis, Microbiome-Gut-Brain[Title/Abstract])) OR (Microbiome Gut Brain Axis[Title/Abstract])) OR (Gut-Brain-Microbiome Axis[Title/Abstract])) OR (Axis, Gut-Brain-Microbiome[Title/Abstract])) OR (Gut Brain Microbiome Axis[Title/Abstract])) OR (Microbiome-Brain-Gut Axis[Title/Abstract])) OR (Axis, Microbiome-Brain-Gut[Title/Abstract])) OR (Microbiome Brain Gut Axis[Title/Abstract])) OR (Microbiota-Brain-Gut Axis[Title/Abstract])) OR (Axis, Microbiota-Brain-Gut[Title/Abstract])) OR (Microbiota Brain Gut Axis[Title/Abstract])) | 4034 |
|  | **#29 Enteric Nervous System** |  |
|  | ("Enteric Nervous System"[Mesh]) OR (((((Enteric Nervous Systems[Title/Abstract]) OR (Nervous System, Enteric[Title/Abstract])) OR (Nervous Systems, Enteric[Title/Abstract])) OR (System, Enteric Nervous[Title/Abstract])) OR (Systems, Enteric Nervous[Title/Abstract])) | 9680 |
|  | **# 30 Gastrointestinal Microbiome** |  |
|  | ("Gastrointestinal Microbiome"[Mesh]) OR ((((((((((((((((((((((((((((((((((((((Gastrointestinal Microbiome[Title/Abstract]) OR (Gastrointestinal Microbiomes[Title/Abstract])) OR (Microbiome, Gastrointestinal[Title/Abstract])) OR (Gut Microbiome[Title/Abstract])) OR (Gut Microbiomes[Title/Abstract])) OR (Microbiome, Gut[Title/Abstract])) OR (Gut Microflora[Title/Abstract])) OR (Microflora, Gut[Title/Abstract])) OR (Gut Microbiota[Title/Abstract])) OR (Gut Microbiotas[Title/Abstract])) OR (Microbiota, Gut[Title/Abstract])) OR (Gastrointestinal Flora[Title/Abstract])) OR (Flora, Gastrointestinal[Title/Abstract])) OR (Gut Flora[Title/Abstract])) OR (Flora, Gut[Title/Abstract])) OR (Gastrointestinal Microbiota[Title/Abstract])) OR (Gastrointestinal Microbiotas[Title/Abstract])) OR (Microbiota, Gastrointestinal[Title/Abstract])) OR (Gastrointestinal Microbial Community[Title/Abstract])) OR (Gastrointestinal Microbial Communities[Title/Abstract])) OR (Microbial Community, Gastrointestinal[Title/Abstract])) OR (Gastrointestinal Microflora[Title/Abstract])) OR (Microflora, Gastrointestinal[Title/Abstract])) OR (Gastric Microbiome[Title/Abstract])) OR (Gastric Microbiomes[Title/Abstract])) OR (Microbiome, Gastric[Title/Abstract])) OR (Intestinal Microbiome[Title/Abstract])) OR (Intestinal Microbiomes[Title/Abstract])) OR (Microbiome, Intestinal[Title/Abstract])) OR (Intestinal Microbiota[Title/Abstract])) OR (Intestinal Microbiotas[Title/Abstract])) OR (Microbiota, Intestinal[Title/Abstract])) OR (Intestinal Microflora[Title/Abstract])) OR (Microflora, Intestinal[Title/Abstract])) OR (Intestinal Flora[Title/Abstract])) OR (Flora, Intestinal[Title/Abstract])) OR (Enteric Bacteria[Title/Abstract])) OR (Bacteria, Enteric[Title/Abstract])) | 68321 |
|  | **# 31: #28 OR #29 OR #30** | 79229 |
|  | **# 32: #27 AND #31** | 27688 |
|  | **Filters applied: Meta-Analysis, Systematic Review.** | **465** |
| The Cochrane library | **#1: Nervous System Diseases** | 164257 |
|  | MeSH descriptor: [Nervous System Diseases] explode all trees OR  (Nervous System Diseases or Disease, Nervous System or Diseases, Nervous System or Nervous System Disease or Neurologic Disorders or Disorder, Neurologic or Disorders, Neurologic or Neurologic Disorder or Neurological Disorders or Disorder, Neurological or Disorders, Neurological or Neurological Disorder or Nervous System Disorders or Disorder, Nervous System or Disorders, Nervous System or Nervous System Disorder):ti,ab,kw |  |
|  | **#2： Neurocognitive Disorders** | 16372 |
|  | MeSH descriptor: [Neurocognitive Disorders] explode all trees OR  (Neurocognitive Disorders or Disorder, Neurocognitive or Disorders, Neurocognitive or Neurocognitive Disorder or Organic Brain Syndrome, Nonpsychotic or Nonpsychotic Organic Brain Syndrome or Organic Mental Disorders, Psychotic or Psychoses, Traumatic or Traumatic Psychoses or Delirium, Dementia, Amnestic, Cognitive Disorders or Mental Disorders, Organic or Disorders, Organic Mental or Mental Disorder, Organic or Organic Mental Disorder or Organic Mental Disorders or Kandinsky Syndrome or Clerambault Syndrome):ti,ab,kw |  |
|  | **#3： Central Nervous System** | 25967 |
|  | MeSH descriptor: [Central Nervous System] explode all trees OR  (Central Nervous System or Central Nervous Systems or Nervous System, Central or Nervous Systems, Central or Systems, Central Nervous or Cerebrospinal Axis or Axi, Cerebrospinal or Axis, Cerebrospinal or Cerebrospinal Axi):ti,ab,kw |  |
|  | **#4: Mental Disorders** | 117800 |
|  | MeSH descriptor: [Mental Disorders] explode all trees OR  (Mental Disorders or Mental Disorder or Psychiatric Illness or Psychiatric Illnesses or Psychiatric Diseases or Psychiatric Disease or Mental Illness or Illness, Mental or Mental Illnesses or Psychiatric Disorders or Psychiatric Disorder or Behavior Disorders or Diagnosis, Psychiatric or Psychiatric Diagnosis or Mental Disorders, Severe or Mental Disorder, Severe or Severe Mental Disorder or Severe Mental Disorders):ti,ab,kw |  |
|  | **#5: Brain Injuries** | 9568 |
|  | MeSH descriptor: [Brain Injuries] explode all trees OR  (Brain Injuries or Injuries, Brain or Brain Injury or Injury, Brain or Injuries, Acute Brain or Acute Brain Injuries or Acute Brain Injury or Brain Injury, Acute or Injury, Acute Brain or Brain Injuries, Acute or Brain Lacerations or Brain Laceration or Laceration, Brain or Lacerations, Brain or Brain Injuries, Focal or Brain Injury, Focal or Focal Brain Injury or Injuries, Focal Brain or Injury, Focal Brain or Focal Brain Injuries):ti,ab,kw |  |
|  | **#6: Parkinson Disease** | 43973 |
|  | MeSH descriptor: [Parkinson Disease] explode all trees OR  (Parkinson disease or Idiopathic Parkinson's Disease or Lewy Body Parkinson's Disease or Parkinson's Disease, Idiopathic or Parkinson's Disease, Lewy Body or Parkinson Disease, Idiopathic or Parkinson's Disease or Idiopathic Parkinson Disease or Lewy Body Parkinson Disease or Primary Parkinsonism or Parkinsonism, Primary or Paralysis Agitans or PD):ti,ab,kw |  |
|  | **#7: Multiple Sclerosis** | 11318 |
|  | MeSH descriptor: [Multiple Sclerosis] explode all trees OR  (Multiple Sclerosis or Sclerosis, Multiple or Sclerosis, Disseminated or Disseminated Sclerosis or MS (Multiple Sclerosis) or Multiple Sclerosis, Acute Fulminating):ti,ab,kw |  |
|  | **#8: Alzheimer's disease** | 12238 |
|  | MeSH descriptor: [Alzheimer Disease] explode all trees OR  (Alzheimer Disease or Alzheimer Dementia or Alzheimer Dementias or Dementia, Alzheimer or Alzheimer's Disease or Dementia, Senile or Senile Dementia or Dementia, Alzheimer Type or Alzheimer Type Dementia or Alzheimer-Type Dementia (ATD) or Alzheimer Type Dementia (ATD) or Dementia, Alzheimer-Type (ATD) or Alzheimer Type Senile Dementia or Primary Senile Degenerative Dementia or Dementia, Primary Senile Degenerative or Alzheimer Sclerosis or Sclerosis, Alzheimer or Alzheimer Syndrome or Alzheimer's Diseases or Alzheimer Diseases or Alzheimers Diseases or Senile Dementia, Alzheimer Type or Acute Confusional Senile Dementia or Senile Dementia, Acute Confusional or Dementia, Presenile or Presenile Dementia or Alzheimer Disease, Late Onset or Late Onset Alzheimer Disease or Alzheimer's Disease, Focal Onset or Focal Onset Alzheimer's Disease or Familial Alzheimer Disease (FAD) or Alzheimer Disease, Familial (FAD) or Familial Alzheimer Diseases (FAD) or Alzheimer Disease, Early Onset or Early Onset Alzheimer Disease or Presenile Alzheimer Dementia):ti,ab,kw |  |
|  | **#9: Brain Ischemia** | 10197 |
|  | MeSH descriptor: [Brain Ischemia] explode all trees OR  (Brain Ischemia or Brain Ischemias or Ischemia, Brain or Ischemic Encephalopathy or Encephalopathy, Ischemic or Ischemic Encephalopathies or Cerebral Ischemia or Cerebral Ischemias or Ischemias, Cerebral or Ischemia, Cerebral):ti,ab,kw |  |
|  | **#10: Intracranial Hemorrhages** | 9334 |
|  | MeSH descriptor: [Intracranial Hemorrhages] explode all trees OR  (Intracranial Hemorrhages or Hemorrhages, Intracranial or Intracranial Hemorrhage or Hemorrhage, Intracranial or Posterior Fossa Hemorrhage or Hemorrhage, Posterior Fossa or Hemorrhages, Posterior Fossa or Posterior Fossa Hemorrhages or Brain Hemorrhage or Brain Hemorrhages or Hemorrhage, Brain or Hemorrhages, Brain):ti,ab,kw |  |
|  | **#11: Hepatic Encephalopathy** | 2159 |
|  | MeSH descriptor: [Hepatic Encephalopathy] explode all trees OR  (Hepatic encephalopathy or Encephalopathies, Hepatic or Hepatic Encephalopathies or Encephalopathy, Hepatic or Portal-Systemic Encephalopathy or Portal Systemic Encephalopathy or Encephalopathy, Portal-Systemic or Encephalopathies, Portal-Systemic or Encephalopathy, Portal Systemic or Portal-Systemic Encephalopathies or Encephalopathy, Portosystemic or Hepatocerebral Encephalopathy or Portosystemic Encephalopathy or Encephalopathies, Portosystemic or Portosystemic Encephalopathies or Encephalopathy, Hepatocerebral or Encephalopathies, Hepatocerebral or Hepatocerebral Encephalopathies or Hepatic Coma or Coma, Hepatic or Comas, Hepatic or Hepatic Comas or Hepatic Stupor or Hepatic Stupors or Stupor, Hepatic or Stupors, Hepatic or Fulminant Hepatic Failure with Cerebral Edema):ti,ab,kw |  |
|  | **#12: Stroke** | 65333 |
|  | MeSH descriptor: [Stroke] explode all trees OR  (Stroke or Strokes or Cerebrovascular Accident or Cerebrovascular Accidents or CVA (Cerebrovascular Accident) or CVAs (Cerebrovascular Accident) or Cerebrovascular Apoplexy or Apoplexy, Cerebrovascular or Vascular Accident, Brain or Brain Vascular Accident or Brain Vascular Accidents or Vascular Accidents, Brain or Cerebrovascular Stroke or Cerebrovascular Strokes or Stroke, Cerebrovascular or Strokes, Cerebrovascular or Apoplexy or Cerebral Stroke or Cerebral Strokes or Stroke, Cerebral or Strokes, Cerebral or Stroke, Acute or Acute Stroke or Acute Strokes or Strokes, Acute or Cerebrovascular Accident, Acute or Acute Cerebrovascular Accident or Acute Cerebrovascular Accidents or Cerebrovascular Accidents, Acute):ti,ab,kw |  |
|  | **#13 Spinal Cord Injuries** | 9787 |
|  | MeSH descriptor: [Spinal Cord Injuries] explode all trees OR  (Spinal Cord Injuries or Spinal Cord Trauma or Cord Trauma, Spinal or Cord Traumas, Spinal or Spinal Cord Traumas or Trauma, Spinal Cord or Traumas, Spinal Cord or Myelopathy, Traumatic or Myelopathies, Traumatic or Traumatic Myelopathies or Traumatic Myelopathy or Injuries, Spinal Cord or Cord Injuries, Spinal or Cord Injury, Spinal or Injury, Spinal Cord or Spinal Cord Injury or Spinal Cord Transection or Cord Transection, Spinal or Cord Transections, Spinal or Spinal Cord Transections or Transection, Spinal Cord or Transections, Spinal Cord or Spinal Cord Laceration or Cord Laceration, Spinal or Cord Lacerations, Spinal or Laceration, Spinal Cord or Lacerations, Spinal Cord or Spinal Cord Lacerations or Post-Traumatic Myelopathy or Myelopathies, Post-Traumatic or Myelopathy, Post-Traumatic or Post Traumatic Myelopathy or Post-Traumatic Myelopathies or Spinal Cord Contusion or Contusion, Spinal Cord or Contusions, Spinal Cord or Cord Contusion, Spinal or Cord Contusions, Spinal or Spinal Cord Contusions):ti,ab,kw |  |
|  | **#14 Traumatic Brain Injury** | 5376 |
|  | MeSH descriptor: [Brain Injuries, Traumatic] explode all trees OR  (Brain Injuries, Traumatic or Brain Injury, Traumatic or Traumatic Brain Injuries or Trauma, Brain or Brain Trauma or Brain Traumas or Traumas, Brain or TBI (Traumatic Brain Injury) or Encephalopathy, Traumatic or Encephalopathies, Traumatic or Traumatic Encephalopathies or Injury, Brain, Traumatic or Traumatic Encephalopathy or TBIs (Traumatic Brain Injuries) or TBI (Traumatic Brain Injuries) or Traumatic Brain Injury):ti,ab,kw |  |
|  | **#15 Amyotrophic Lateral Sclerosis** | 1848 |
|  | MeSH descriptor: [Amyotrophic Lateral Sclerosis] explode all trees OR  (Amyotrophic Lateral Sclerosis or Sclerosis, Amyotrophic Lateral or Gehrig's Disease or Gehrig Disease or Gehrigs Disease or Charcot Disease or Motor Neuron Disease, Amyotrophic Lateral Sclerosis or Lou Gehrig's Disease or Lou Gehrigs Disease or Disease, Lou Gehrig's or ALS Amyotrophic Lateral Sclerosis or ALS Amyotrophic Lateral Sclerosis or Lou Gehrig Disease or Amyotrophic Lateral Sclerosis, Guam Form or Amyotrophic Lateral Sclerosis-Parkinsonism-Dementia Complex 1 or Amyotrophic Lateral Sclerosis Parkinsonism Dementia Complex 1 or Guam Form of Amyotrophic Lateral Sclerosis or Guam Disease or Disease, Guam or Amyotrophic Lateral Sclerosis, Parkinsonism-Dementia Complex of Guam or Amyotrophic Lateral Sclerosis, Parkinsonism Dementia Complex of Guam or Amyotrophic Lateral Sclerosis With Dementia or Dementia With Amyotrophic Lateral Sclerosis) :ti,ab,kw |  |
|  | **#16 Epilepsy** | 10928 |
|  | MeSH descriptor: [Epilepsy] explode all trees OR  (Epilepsy or Epilepsies or Seizure Disorder or Seizure Disorders or Awakening Epilepsy or Epilepsy, Awakening or Epilepsy, Cryptogenic or Cryptogenic Epilepsies or Cryptogenic Epilepsy or Epilepsies, Cryptogenic or Aura or Auras):ti,ab,kw |  |
|  | **#17 Autism Spectrum Disorder** | 3297 |
|  | MeSH descriptor: [Autism Spectrum Disorder] explode all trees OR  (Autism Spectrum Disorder or Autism Spectrum Disorders or Autistic Spectrum Disorder or Autistic Spectrum Disorders or Disorder, Autistic Spectrum):ti,ab,kw |  |
|  | **#18 Attention Deficit Disorder with Hyperactivity** | 8007 |
|  | MeSH descriptor: [Attention Deficit Disorder with Hyperactivity] explode all trees OR  (Attention Deficit Disorder with Hyperactivity or Attention Deficit Disorders with Hyperactivity or ADHD or Attention Deficit Hyperactivity Disorder or Hyperkinetic Syndrome or Syndromes, Hyperkinetic or Attention Deficit-Hyperactivity Disorder or Attention Deficit-Hyperactivity Disorders or Deficit-Hyperactivity Disorder, Attention or Deficit-Hyperactivity Disorders, Attention or Disorder, Attention Deficit-Hyperactivity or Disorders, Attention Deficit-Hyperactivity or ADDH or Attention Deficit Hyperactivity Disorders or Attention Deficit Disorder or Attention Deficit Disorders or Deficit Disorder, Attention or Deficit Disorders, Attention or Disorder, Attention Deficit or Disorders, Attention Deficit or Brain Dysfunction, Minimal or Dysfunction, Minimal Brain or Minimal Brain Dysfunction):ti,ab,kw | 3 |
|  | **#19 Hyperkinesis** | 1275 |
|  | MeSH descriptor: [Hyperkinesis] explode all trees OR  (Hyperkinesis or Hyperkinesia or Hyperkinetic Movements or Hyperkinetic Movement or Movement, Hyperkinetic or Movements, Hyperkinetic or Hyperactivity, Motor or Motor Hyperactivity or Hyperkinesia, Generalized or Generalized Hyperkinesia or Generalized Hyperkinesias or Hyperkinesias, Generalized):ti,ab,kw |  |
|  | **# 20 schizophrenia** | 17887 |
|  | MeSH descriptor: [Schizophrenia] explode all trees OR  (schizophrenia or Schizophrenias or Schizophrenic Disorders or Disorder, Schizophrenic or Disorders, Schizophrenic or Schizophrenic Disorder or Dementia Praecox):ti,ab,kw |  |
|  | **# 21 bipolar disorder** | 7485 |
|  | MeSH descriptor: [Bipolar Disorder] explode all trees OR  (bipolar disorder or Bipolar Disorders or Disorder, Bipolar or Affective Psychosis, Bipolar or Bipolar Affective Psychosis or Psychoses, Bipolar Affective or Psychosis, Bipolar Affective or Manic-Depressive Psychosis or Manic Depressive Psychosis or Psychosis, Manic-Depressive or Psychosis, Manic Depressive or Bipolar Mood Disorder or Bipolar Mood Disorders or Disorder, Bipolar Mood or Mood Disorder, Bipolar or Psychoses, Manic-Depressive or Psychoses, Manic Depressive or Depression, Bipolar or Bipolar Depression or Manic Depression or Depression, Manic or Depressions, Manic or Manic Disorder or Disorder, Manic or Manic Disorders):ti,ab,kw |  |
|  | **# 22 Cognitive Dysfunction** | 23102 |
|  | MeSH descriptor: [Cognitive Dysfunction] explode all trees OR  (Cognitive Dysfunction or Cognitive Dysfunctions or Dysfunction, Cognitive or Dysfunctions, Cognitive or Cognitive Impairments or Cognitive Impairment or Impairment, Cognitive or Impairments, Cognitive or Mild Cognitive Impairment or Cognitive Impairment, Mild or Cognitive Impairments, Mild or Impairment, Mild Cognitive or Impairments, Mild Cognitive or Mild Cognitive Impairments or Mild Neurocognitive Disorder or Disorder, Mild Neurocognitive or Disorders, Mild Neurocognitive or Mild Neurocognitive Disorders or Neurocognitive Disorder, Mild or Neurocognitive Disorders, Mild or Cognitive Decline or Cognitive Declines or Decline, Cognitive or Declines, Cognitive or Mental Deterioration or Deterioration, Mental or Deteriorations, Mental or Mental Deteriorations):ti,ab,kw |  |
|  | **# 23 Alexithymia** | 3545 |
|  | MeSH descriptor: [Affective Symptoms] explode all trees OR  (Affective Symptoms or Affective Symptom or Symptom, Affective or Symptoms, Affective or Alexithymia or Alexithymias or Emotional Disturbances or Disturbance, Emotional or Disturbances, Emotional or Emotional Disturbance):ti,ab,kw |  |
|  | **# 24 Depressive Disorder** | 26495 |
|  | MeSH descriptor: [Depressive Disorder] explode all trees OR  (Depressive Disorder or Depressive Disorders or Disorder, Depressive or Disorders, Depressive or Neurosis, Depressive or Depressive Neuroses or Depressive Neurosis or Neuroses, Depressive or Depression, Endogenous or Depressions, Endogenous or Endogenous Depression or Endogenous Depressions or Depressive Syndrome or Depressive Syndromes or Syndrome, Depressive or Syndromes, Depressive or Depression, Neurotic or Depressions, Neurotic or Neurotic Depression or Neurotic Depressions or Melancholia or Melancholias or Unipolar Depression or Depression, Unipolar or Depressions, Unipolar or Unipolar Depressions):ti,ab,kw |  |
|  | **# 25 Dementia** | 14509 |
|  | MeSH descriptor: [Dementia] explode all trees OR  (Dementia or Dementias or Amentia or Amentias or Senile Paranoid Dementia or Dementias, Senile Paranoid or Paranoid Dementia, Senile or Paranoid Dementias, Senile or Senile Paranoid Dementias or Familial Dementia or Dementia, Familial or Dementias, Familial or Familial Dementias):ti,ab,kw |  |
|  | # **26 Anxiety Disorders** | 27389 |
|  | MeSH descriptor: [Anxiety Disorders] explode all trees OR  (Anxiety Disorders or Anxiety Disorder or Disorder, Anxiety or Disorders, Anxiety or Neuroses, Anxiety or Anxiety Neuroses or Anxiety States, Neurotic or Anxiety State, Neurotic or Neurotic Anxiety State or Neurotic Anxiety States or State, Neurotic Anxiety or States, Neurotic Anxiety):ti,ab,kw |  |
|  | **# 27 : #1 OR #2 OR #3 OR #4 OR #5 OR #6 OR #7 OR #8 OR #9 OR #10 OR #11 OR #12 OR #13 OR #14 OR #15 OR #16 OR #17 OR #18 OR #19 OR #20 OR #21 OR #22 OR #23 OR #24 OR #25 OR #26** | 434807 |
|  | **#28 Gut-Brain Axis** | 288 |
|  | MeSH descriptor: [Brain-Gut Axis] explode all trees OR  (Gut-Brain Axis or Axis, Brain-Gut or Brain Gut Axis or Gut and Brain Axis or Gut-Brain Axis or Axis, Gut-Brain or Gut Brain Axis or Brain and Gut Axis or Microbiota-Gut-Brain Axis or Axis, Microbiota-Gut-Brain or Microbiota Gut Brain Axis or Brain-Gut-Microbiome Axis or Axis, Brain-Gut-Microbiome or Brain Gut Microbiome Axis or Microbiome-Gut-Brain Axis or Axis, Microbiome-Gut-Brain or Microbiome Gut Brain Axis or Gut-Brain-Microbiome Axis or Axis, Gut-Brain-Microbiome or Gut Brain Microbiome Axis or Microbiome-Brain-Gut Axis or Axis, Microbiome-Brain-Gut or Microbiome Brain Gut Axis or Microbiota-Brain-Gut Axis or Axis, Microbiota-Brain-Gut or Microbiota Brain Gut Axis):ti,ab,kw |  |
|  | **#29 Enteric Nervous System** | 97 |
|  | MeSH descriptor: [Enteric Nervous System] explode all trees OR  (Enteric Nervous Systems or Nervous System, Enteric or Nervous Systems, Enteric or System, Enteric Nervous or Systems, Enteric Nervous):ti,ab,kw |  |
|  | **# 30 Gastrointestinal Microbiome** | 7186 |
|  | MeSH descriptor: [Gastrointestinal Microbiome] explode all trees OR  (Gastrointestinal Microbiome or Gastrointestinal Microbiomes or Microbiome, Gastrointestinal or Gut Microbiome or Gut Microbiomes or Microbiome, Gut or Gut Microflora or Microflora, Gut or Gut Microbiota or Gut Microbiotas or Microbiota, Gut or Gastrointestinal Flora or Flora, Gastrointestinal or Gut Flora or Flora, Gut or Gastrointestinal Microbiota or Gastrointestinal Microbiotas or Microbiota, Gastrointestinal or Gastrointestinal Microbial Community or Gastrointestinal Microbial Communities or Microbial Community, Gastrointestinal or Gastrointestinal Microflora or Microflora, Gastrointestinal or Gastric Microbiome or Gastric Microbiomes or Microbiome, Gastric or Intestinal Microbiome or Intestinal Microbiomes or Microbiome, Intestinal or Intestinal Microbiota or Intestinal Microbiotas or Microbiota, Intestinal or Intestinal Microflora or Microflora, Intestinal or Intestinal Flora or Flora, Intestinal or Enteric Bacteria or Bacteria, Enteric):ti,ab,kw |  |
|  | **# 31: #28 OR #29 OR #30** | 7401 |
|  | **# 32: #27 AND #31** | 1336 |
|  | Filters applied: Meta-Analysis, Systematic Review. | 27 |
| **Embase** | **#1: Nervous System Diseases** | 4506567 |
|  | 'nervous system diseases'/exp OR  'nervous system diseases':ab,ti OR 'disease, nervous system':ab,ti OR 'diseases, nervous system':ab,ti OR 'nervous system disease':ab,ti OR 'neurologic disorders':ab,ti OR 'disorder, neurologic':ab,ti OR 'disorders, neurologic':ab,ti OR 'neurologic disorder':ab,ti OR 'neurological disorders':ab,ti OR 'disorder, neurological':ab,ti OR 'disorders, neurological':ab,ti OR 'neurological disorder':ab,ti OR 'nervous system disorders':ab,ti OR 'disorder, nervous system':ab,ti OR 'disorders, nervous system':ab,ti OR 'nervous system disorder':ab,ti |  |
|  | **#2： Neurocognitive Disorders** | 928214 |
|  | 'disorders of higher cerebral function'/exp OR  'neurocognitive disorders':ab,ti OR 'disorder, neurocognitive':ab,ti OR 'disorders, neurocognitive':ab,ti OR 'neurocognitive disorder':ab,ti OR 'organic brain syndrome, nonpsychotic':ab,ti OR 'nonpsychotic organic brain syndrome':ab,ti OR 'organic mental disorders, psychotic':ab,ti OR 'psychoses, traumatic':ab,ti OR 'traumatic psychoses':ab,ti OR 'delirium, dementia, amnestic, cognitive disorders':ab,ti OR 'mental disorders, organic':ab,ti OR 'neurological disorder':ab,ti OR 'disorders, organic mental':ab,ti OR 'mental disorder, organic':ab,ti OR 'organic mental disorder':ab,ti OR 'organic mental disorders':ab,ti OR 'kandinsky syndrome':ab,ti OR 'clerambault syndrome':ab,ti |  |
|  | **#3： Central Nervous System** | 2310826 |
|  | 'central nervous system'/exp OR  'central nervous system':ab,ti OR 'central nervous systems':ab,ti OR 'nervous system, central':ab,ti OR 'nervous systems, central':ab,ti OR 'systems, central nervous':ab,ti OR 'cerebrospinal axis':ab,ti OR 'axi, cerebrospinal':ab,ti OR 'axis, cerebrospinal':ab,ti OR 'cerebrospinal axi':ab,ti |  |
|  | **#4: Mental Disorders** | 2,581,107 |
|  | 'mental disorders'/exp OR  (Mental Disorders or Mental Disorder or Psychiatric Illness or Psychiatric Illnesses or Psychiatric Diseases or Psychiatric Disease or Mental Illness or Illness, Mental or Mental Illnesses or Psychiatric Disorders or Psychiatric Disorder or Behavior Disorders or Diagnosis, Psychiatric or Psychiatric Diagnosis or Mental Disorders, Severe or Mental Disorder, Severe or Severe Mental Disorder or Severe Mental Disorders):ti,ab,kw |  |
|  | **#5: Brain Injuries** | 205150 |
|  | 'brain injuries'/exp OR  'brain injuries':ab,ti OR 'injuries, brain':ab,ti OR 'brain injury':ab,ti OR 'injury, brain':ab,ti OR 'injuries, acute brain':ab,ti OR 'acute brain injuries':ab,ti OR 'acute brain injury':ab,ti OR 'brain injury, acute':ab,ti OR 'injury, acute brain':ab,ti OR 'brain injuries, acute':ab,ti OR 'brain lacerations':ab,ti OR 'brain laceration':ab,ti OR 'laceration, brain':ab,ti OR 'lacerations, brain':ab,ti OR 'brain injuries, focal':ab,ti OR 'brain injury, focal':ab,ti OR 'focal brain injury':ab,ti OR 'injuries, focal brain':ab,ti OR 'injury, focal brain':ab,ti OR 'focal brain injuries':ab,ti |  |
|  | **#6: Parkinson Disease** | 371666 |
|  | 'parkinson disease'/exp OR  'parkinson disease, lewy body':ab,ti OR 'parkinson disease, idiopathic':ab,ti OR 'parkinson disease':ab,ti OR 'idiopathic parkinson disease':ab,ti OR 'lewy body parkinson disease':ab,ti OR 'primary parkinsonism':ab,ti OR 'parkinsonism, primary':ab,ti OR 'paralysis agitans':ab,ti OR 'pd':ab,ti |  |
|  | **#7: Multiple Sclerosis** | 161583 |
|  | 'multiple sclerosis'/exp OR  'multiple sclerosis':ab,ti OR 'sclerosis, multiple':ab,ti OR 'sclerosis, disseminated':ab,ti OR 'disseminated sclerosis':ab,ti OR 'ms (multiple sclerosis)':ab,ti OR 'multiple sclerosis, acute fulminating':ab,ti |  |
|  | **#8: Alzheimer's disease** | 230709 |
|  | 'alzheimer disease'/exp OR  'alzheimer dementia':ab,ti OR 'alzheimer dementias':ab,ti OR 'dementia, alzheimer':ab,ti OR 'alzheimer disease':ab,ti OR 'dementia, senile':ab,ti OR 'senile dementia':ab,ti OR 'dementia, alzheimer type':ab,ti OR 'alzheimer type dementia':ab,ti OR 'alzheimer-type dementia (atd)':ab,ti OR 'alzheimer type dementia (atd)':ab,ti OR 'dementia, alzheimer-type (atd)':ab,ti OR 'alzheimer type senile dementia':ab,ti OR 'primary senile degenerative dementia':ab,ti OR 'dementia, primary senile degenerative':ab,ti OR 'alzheimer sclerosis':ab,ti OR 'sclerosis, alzheimer':ab,ti OR 'alzheimer syndrome':ab,ti OR 'alzheimer diseases':ab,ti OR 'alzheimers diseases':ab,ti OR 'senile dementia, alzheimer type':ab,ti OR 'acute confusional senile dementia':ab,ti OR 'senile dementia, acute confusional':ab,ti OR 'dementia, presenile':ab,ti OR 'presenile dementia':ab,ti OR 'alzheimer disease, late onset':ab,ti OR 'late onset alzheimer disease':ab,ti OR 'alzheimer disease, focal onset':ab,ti OR 'focal onset alzheimer disease':ab,ti OR 'familial alzheimer disease (fad)':ab,ti OR 'alzheimer disease, familial (fad)':ab,ti OR 'familial alzheimer diseases (fad)':ab,ti OR 'alzheimer disease, early onset':ab,ti OR 'early onset alzheimer disease':ab,ti OR 'presenile alzheimer dementia':ab,ti |  |
|  | **#9: Brain Ischemia** | 215025 |
|  | 'brain ischemia'/exp OR  'brain ischemia':ab,ti OR 'brain ischemias':ab,ti OR 'ischemia, brain':ab,ti OR 'ischemic encephalopathy':ab,ti OR 'encephalopathy, ischemic':ab,ti OR 'ischemic encephalopathies':ab,ti OR 'cerebral ischemia':ab,ti OR 'cerebral ischemias':ab,ti OR 'ischemias, cerebral':ab,ti OR 'ischemia, cerebral':ab,ti |  |
|  | **#10: Intracranial Hemorrhages** | 169732 |
|  | 'intracranial hemorrhages'/exp OR  'intracranial hemorrhages':ab,ti OR 'hemorrhages, intracranial':ab,ti OR 'intracranial hemorrhage':ab,ti OR 'hemorrhage, intracranial':ab,ti OR 'posterior fossa hemorrhage':ab,ti OR 'hemorrhage, posterior fossa':ab,ti OR 'hemorrhages, posterior fossa':ab,ti OR 'posterior fossa hemorrhages':ab,ti OR 'brain hemorrhage':ab,ti OR 'brain hemorrhages':ab,ti OR 'hemorrhage, brain':ab,ti OR 'hemorrhages, brain':ab,ti |  |
|  | **#11: Hepatic Encephalopathy** | 26553 |
|  | 'hepatic encephalopathy'/exp OR  'hepatic encephalopathy':ab,ti OR 'encephalopathies, hepatic':ab,ti OR 'hepatic encephalopathies':ab,ti OR 'encephalopathy, hepatic':ab,ti OR 'portal-systemic encephalopathy':ab,ti OR 'portal systemic encephalopathy':ab,ti OR 'encephalopathy, portal-systemic':ab,ti OR 'encephalopathies, portal-systemic':ab,ti OR 'encephalopathy, portal systemic':ab,ti OR 'portal-systemic encephalopathies':ab,ti OR 'encephalopathy, portosystemic':ab,ti OR 'hepatocerebral encephalopathy':ab,ti OR 'portosystemic encephalopathy':ab,ti OR 'encephalopathies, portosystemic':ab,ti OR 'portosystemic encephalopathies':ab,ti OR 'encephalopathy, hepatocerebral':ab,ti OR 'encephalopathies, hepatocerebral':ab,ti OR 'hepatocerebral encephalopathies':ab,ti OR 'hepatic coma':ab,ti OR 'coma, hepatic':ab,ti OR 'comas, hepatic':ab,ti OR 'hepatic comas':ab,ti OR 'hepatic stupor':ab,ti OR 'hepatic stupors':ab,ti OR 'stupor, hepatic':ab,ti OR 'stupors, hepatic':ab,ti OR 'fulminant hepatic failure with cerebral edema':ab,ti |  |
|  | **#12: Stroke** | 564905 |
|  | 'stroke'/exp OR  'stroke':ab,ti OR 'strokes':ab,ti OR 'cerebrovascular accident':ab,ti OR 'cerebrovascular accidents':ab,ti OR 'cva (cerebrovascular accident)':ab,ti OR 'cvas (cerebrovascular accident)':ab,ti OR 'cerebrovascular apoplexy':ab,ti OR 'apoplexy, cerebrovascular':ab,ti OR 'vascular accident, brain':ab,ti OR 'brain vascular accident':ab,ti OR 'brain vascular accidents':ab,ti OR 'vascular accidents, brain':ab,ti OR 'cerebrovascular stroke':ab,ti OR 'cerebrovascular strokes':ab,ti OR 'stroke, cerebrovascular':ab,ti OR 'strokes, cerebrovascular':ab,ti OR 'apoplexy':ab,ti OR 'cerebral stroke':ab,ti OR 'cerebral strokes':ab,ti OR 'stroke, cerebral':ab,ti OR 'strokes, cerebral':ab,ti OR 'stroke, acute':ab,ti OR 'acute stroke':ab,ti OR 'acute strokes':ab,ti OR 'strokes, acute':ab,ti OR 'cerebrovascular accident, acute':ab,ti OR 'acute cerebrovascular accident':ab,ti OR 'acute cerebrovascular accidents':ab,ti OR 'cerebrovascular accidents, acute':ab,ti |  |
|  | **#13 Spinal Cord Injuries** | 94359 |
|  | 'spinal cord injuries'/exp OR  'spinal cord injuries':ab,ti OR 'spinal cord trauma':ab,ti OR 'cord trauma, spinal':ab,ti OR 'cord traumas, spinal':ab,ti OR 'spinal cord traumas':ab,ti OR 'trauma, spinal cord':ab,ti OR 'traumas, spinal cord':ab,ti OR 'myelopathy, traumatic':ab,ti OR 'myelopathies, traumatic':ab,ti OR 'traumatic myelopathies':ab,ti OR 'traumatic myelopathy':ab,ti OR 'injuries, spinal cord':ab,ti OR 'cord injuries, spinal':ab,ti OR 'cord injury, spinal':ab,ti OR 'injury, spinal cord':ab,ti OR 'spinal cord injury':ab,ti OR 'spinal cord transection':ab,ti OR 'cord transection, spinal':ab,ti OR 'cord transections, spinal':ab,ti OR 'spinal cord transections':ab,ti OR 'transection, spinal cord':ab,ti OR 'transections, spinal cord':ab,ti OR 'spinal cord laceration':ab,ti OR 'cord laceration, spinal':ab,ti OR 'cord lacerations, spinal':ab,ti OR 'laceration, spinal cord':ab,ti OR 'lacerations, spinal cord':ab,ti OR 'spinal cord lacerations':ab,ti OR 'post-traumatic myelopathy':ab,ti OR 'myelopathies, post-traumatic':ab,ti OR 'myelopathy, post-traumatic':ab,ti OR 'post traumatic myelopathy':ab,ti OR 'post-traumatic myelopathies':ab,ti OR 'spinal cord contusion':ab,ti OR 'contusion, spinal cord':ab,ti OR 'contusions, spinal cord':ab,ti OR 'cord contusion, spinal':ab,ti OR 'cord contusions, spinal':ab,ti OR 'spinal cord contusions':ab,ti |  |
|  | **#14 Traumatic Brain Injury** | 76,146 |
|  | 'traumatic brain injury'/exp OR  'brain injuries, traumatic':ab,ti OR 'brain injury, traumatic':ab,ti OR 'traumatic brain injuries':ab,ti OR 'trauma, brain':ab,ti OR 'brain trauma':ab,ti OR 'brain traumas':ab,ti OR 'traumas, brain':ab,ti OR 'tbi (traumatic brain injury)':ab,ti OR 'encephalopathy, traumatic':ab,ti OR 'encephalopathies, traumatic':ab,ti OR 'traumatic encephalopathies':ab,ti OR 'injury, brain, traumatic':ab,ti OR 'traumatic encephalopathy':ab,ti OR 'tbis (traumatic brain injuries)':ab,ti OR 'tbi (traumatic brain injuries)':ab,ti OR 'traumatic brain injury':ab,ti |  |
|  | **#15 Amyotrophic Lateral Sclerosis** | 48413 |
|  | 'amyotrophic lateral sclerosis'/exp OR  'amyotrophic lateral sclerosis':ab,ti OR 'sclerosis, amyotrophic lateral':ab,ti OR 'gehrig disease':ab,ti OR 'gehrigs disease':ab,ti OR 'charcot disease':ab,ti OR 'motor neuron disease, amyotrophic lateral sclerosis':ab,ti OR 'lou-gehrigs disease':ab,ti OR 'disease, lou-gehrigs':ab,ti OR 'als - amyotrophic lateral sclerosis':ab,ti OR 'als amyotrophic lateral sclerosis':ab,ti OR 'lou gehrig disease':ab,ti OR 'amyotrophic lateral sclerosis, guam form':ab,ti OR 'amyotrophic lateral sclerosis-parkinsonism-dementia complex 1':ab,ti OR 'amyotrophic lateral sclerosis parkinsonism dementia complex 1':ab,ti OR 'guam form of amyotrophic lateral sclerosis':ab,ti OR 'guam disease':ab,ti OR 'disease, guam':ab,ti OR 'amyotrophic lateral sclerosis, parkinsonism-dementia complex of guam':ab,ti OR 'amyotrophic lateral sclerosis, parkinsonism dementia complex of guam':ab,ti OR 'amyotrophic lateral sclerosis with dementia':ab,ti OR 'dementia with amyotrophic lateral sclerosis':ab,ti |  |
|  | **#16 Epilepsy** | 310592 |
|  | 'epilepsy'/exp OR  'epilepsy':ab,ti OR 'epilepsies':ab,ti OR 'seizure disorder':ab,ti OR 'seizure disorders':ab,ti OR 'awakening epilepsy':ab,ti OR 'epilepsy, awakening':ab,ti OR 'epilepsy, cryptogenic':ab,ti OR 'cryptogenic epilepsies':ab,ti OR 'cryptogenic epilepsy':ab,ti OR 'epilepsies, cryptogenic':ab,ti OR 'aura':ab,ti OR 'auras':ab,ti |  |
|  | **#17 Autism Spectrum Disorder** | 89475 |
|  | 'autism spectrum disorder'/exp OR  'autism spectrum disorder':ab,ti OR 'autism spectrum disorders':ab,ti OR 'autistic spectrum disorder':ab,ti OR 'autistic spectrum disorders':ab,ti OR 'disorder, autistic spectrum':ab,ti |  |
|  | **#18 Attention Deficit Disorder with Hyperactivity** | 76704 |
|  | 'attention deficit disorder with hyperactivity'/exp OR  'attention deficit disorder with hyperactivity':ab,ti OR 'attention deficit disorders with hyperactivity':ab,ti OR 'adhd':ab,ti OR 'attention deficit hyperactivity disorder':ab,ti OR 'hyperkinetic syndrome':ab,ti OR 'syndromes, hyperkinetic':ab,ti OR 'attention deficit-hyperactivity disorder':ab,ti OR 'attention deficit-hyperactivity disorders':ab,ti OR 'deficit-hyperactivity disorder, attention':ab,ti OR 'deficit-hyperactivity disorders, attention':ab,ti OR 'disorder, attention deficit-hyperactivity':ab,ti OR 'disorders, attention deficit-hyperactivity':ab,ti OR 'addh':ab,ti OR 'attention deficit hyperactivity disorders':ab,ti OR 'attention deficit disorder':ab,ti OR 'attention deficit disorders':ab,ti OR 'deficit disorder, attention':ab,ti OR 'deficit disorders, attention':ab,ti OR 'disorder, attention deficit':ab,ti OR 'disorders, attention deficit':ab,ti OR 'brain dysfunction, minimal':ab,ti OR 'dysfunction, minimal brain':ab,ti OR 'minimal brain dysfunction':ab,ti |  |
|  | **#19 Hyperkinesis** | 7569 |
|  | 'hyperkinesis'/exp OR  'hyperkinesis':ab,ti OR 'hyperkinesia':ab,ti OR 'hyperkinetic movements':ab,ti OR 'hyperkinetic movement':ab,ti OR 'movement, hyperkinetic':ab,ti OR 'movements, hyperkinetic':ab,ti OR 'hyperactivity, motor':ab,ti OR 'motor hyperactivity':ab,ti OR 'hyperkinesia, generalized':ab,ti OR 'generalized hyperkinesia':ab,ti OR 'generalized hyperkinesias':ab,ti OR 'hyperkinesias, generalized':ab,ti |  |
|  | **# 20 schizophrenia** | 230957 |
|  | 'schizophrenia'/exp OR  'schizophrenia':ab,ti OR 'schizophrenias':ab,ti OR 'schizophrenic disorders':ab,ti OR 'disorder, schizophrenic':ab,ti OR 'disorders, schizophrenic':ab,ti OR 'schizophrenic disorder':ab,ti OR 'dementia praecox':ab,ti |  |
|  | **# 21 bipolar disorder** | 88269 |
|  | 'bipolar disorder'/exp OR  'bipolar disorder':ab,ti OR 'bipolar disorders':ab,ti OR 'disorder, bipolar':ab,ti OR 'affective psychosis, bipolar':ab,ti OR 'bipolar affective psychosis':ab,ti OR 'psychoses, bipolar affective':ab,ti OR 'psychosis, bipolar affective':ab,ti OR 'manic-depressive psychosis':ab,ti OR 'manic depressive psychosis':ab,ti OR 'psychosis, manic-depressive':ab,ti OR 'psychosis, manic depressive':ab,ti OR 'bipolar mood disorder':ab,ti OR 'bipolar mood disorders':ab,ti OR 'disorder, bipolar mood':ab,ti OR 'mood disorder, bipolar':ab,ti OR 'psychoses, manic-depressive':ab,ti OR 'psychoses, manic depressive':ab,ti OR 'depression, bipolar':ab,ti OR 'bipolar depression':ab,ti OR 'manic depression':ab,ti OR 'depression, manic':ab,ti OR 'depressions, manic':ab,ti OR 'manic disorder':ab,ti OR 'disorder, manic':ab,ti OR 'manic disorders':ab,ti |  |
|  | **# 22 Cognitive Dysfunction** | 588656 |
|  | 'cognitive dysfunction'/exp OR  'cognitive dysfunction':ab,ti OR 'cognitive dysfunctions':ab,ti OR 'dysfunction, cognitive':ab,ti OR 'dysfunctions, cognitive':ab,ti OR 'cognitive impairments':ab,ti OR 'cognitive impairment':ab,ti OR 'impairment, cognitive':ab,ti OR 'impairments, cognitive':ab,ti OR 'mild cognitive impairment':ab,ti OR 'cognitive impairment, mild':ab,ti OR 'cognitive impairments, mild':ab,ti OR 'impairment, mild cognitive':ab,ti OR 'impairments, mild cognitive':ab,ti OR 'mild cognitive impairments':ab,ti OR 'mild neurocognitive disorder':ab,ti OR 'disorder, mild neurocognitive':ab,ti OR 'disorders, mild neurocognitive':ab,ti OR 'mild neurocognitive disorders':ab,ti OR 'neurocognitive disorder, mild':ab,ti OR 'neurocognitive disorders, mild':ab,ti OR 'cognitive decline':ab,ti OR 'cognitive declines':ab,ti OR 'decline, cognitive':ab,ti OR 'declines, cognitive':ab,ti OR 'mental deterioration':ab,ti OR 'deterioration, mental':ab,ti OR 'deteriorations, mental':ab,ti OR 'mental deteriorations':ab,ti |  |
|  | **# 23 Alexithymia** | 11236 |
|  | 'alexithymia'/exp OR  'affective symptoms':ab,ti OR 'affective symptom':ab,ti OR 'symptom, affective':ab,ti OR 'symptoms, affective':ab,ti OR 'alexithymia':ab,ti OR 'alexithymias':ab,ti OR 'emotional disturbances':ab,ti OR 'disturbance, emotional':ab,ti OR 'disturbances, emotional':ab,ti OR 'emotional disturbance':ab,ti |  |
|  | **# 24 Depressive Disorder** | 573823 |
|  | 'depressive disorder'/exp OR  'depressive disorder':ab,ti OR 'depressive disorders':ab,ti OR 'disorder, depressive':ab,ti OR 'disorders, depressive':ab,ti OR 'neurosis, depressive':ab,ti OR 'depressive neuroses':ab,ti OR 'depressive neurosis':ab,ti OR 'neuroses, depressive':ab,ti OR 'depression, endogenous':ab,ti OR 'depressions, endogenous':ab,ti OR 'endogenous depression':ab,ti OR 'endogenous depressions':ab,ti OR 'depressive syndrome':ab,ti OR 'depressive syndromes':ab,ti OR 'syndrome, depressive':ab,ti OR 'syndromes, depressive':ab,ti OR 'depression, neurotic':ab,ti OR 'depressions, neurotic':ab,ti OR 'neurotic depression':ab,ti OR 'neurotic depressions':ab,ti OR 'melancholia':ab,ti OR 'melancholias':ab,ti OR 'unipolar depression':ab,ti OR 'depression, unipolar':ab,ti OR 'depressions, unipolar':ab,ti OR 'unipolar depressions':ab,ti |  |
|  | **# 25 Dementia** | 435274 |
|  | 'dementia'/exp OR  'dementia':ab,ti OR 'dementias':ab,ti OR 'amentia':ab,ti OR 'amentias':ab,ti OR 'senile paranoid dementia':ab,ti OR 'dementias, senile paranoid':ab,ti OR 'paranoid dementia, senile':ab,ti OR 'paranoid dementias, senile':ab,ti OR 'senile paranoid dementias':ab,ti OR 'familial dementia':ab,ti OR 'dementia, familial':ab,ti OR 'dementias, familial':ab,ti OR 'familial dementias':ab,ti |  |
|  | # **26 Anxiety Disorders** | 298755 |
|  | 'anxiety disorders'/exp OR  'anxiety disorders':ab,ti OR 'anxiety disorder':ab,ti OR 'disorder, anxiety':ab,ti OR 'disorders, anxiety':ab,ti OR 'neuroses, anxiety':ab,ti OR 'anxiety neuroses':ab,ti OR 'anxiety states, neurotic':ab,ti OR 'anxiety state, neurotic':ab,ti OR 'neurotic anxiety state':ab,ti OR 'neurotic anxiety states':ab,ti OR 'state, neurotic anxiety':ab,ti OR 'states, neurotic anxiety':ab,ti |  |
|  | **# 27 : #1 OR #2 OR #3 OR #4 OR #5 OR #6 OR #7 OR #8 OR #9 OR #10 OR #11 OR #12 OR #13 OR #14 OR #15 OR #16 OR #17 OR #18 OR #19 OR #20 OR #21 OR #22 OR #23 OR #24 OR #25 OR #26** | 7456517 |
|  | **#28 Gut-Brain Axis** | 4221 |
|  | 'brain-gut axis'/exp OR  'axis, brain-gut':ab,ti OR 'brain gut axis':ab,ti OR 'gut and brain axis':ab,ti OR 'gut-brain axis':ab,ti OR 'axis, gut-brain':ab,ti OR 'gut brain axis':ab,ti OR 'brain and gut axis':ab,ti OR 'microbiota-gut-brain axis':ab,ti OR 'axis, microbiota-gut-brain':ab,ti OR 'microbiota gut brain axis':ab,ti OR 'brain-gut-microbiome axis':ab,ti OR 'axis, brain-gut-microbiome':ab,ti OR 'brain gut microbiome axis':ab,ti OR 'microbiome-gut-brain axis':ab,ti OR 'axis, microbiome-gut-brain':ab,ti OR 'microbiome gut brain axis':ab,ti OR 'gut-brain-microbiome axis':ab,ti OR 'axis, gut-brain-microbiome':ab,ti OR 'gut brain microbiome axis':ab,ti OR 'microbiome-brain-gut axis':ab,ti OR 'axis, microbiome-brain-gut':ab,ti OR 'microbiome brain gut axis':ab,ti OR 'microbiota-brain-gut axis':ab,ti OR 'axis, microbiota-brain-gut':ab,ti OR 'microbiota brain gut axis':ab,ti |  |
|  | **#29 Enteric Nervous System** | 8171 |
|  | 'enteric nervous system'/exp OR  'enteric nervous systems':ab,ti OR 'nervous system, enteric':ab,ti OR 'nervous systems, enteric':ab,ti OR 'system, enteric nervous':ab,ti OR 'systems, enteric nervous':ab,ti |  |
|  | **# 30 Gastrointestinal Microbiome** | 100183 |
|  | 'gastrointestinal microbiome'/exp OR  'gastrointestinal microbiome':ab,ti OR 'gastrointestinal microbiomes':ab,ti OR 'microbiome, gastrointestinal':ab,ti OR 'gut microbiome':ab,ti OR 'gut microbiomes':ab,ti OR 'microbiome, gut':ab,ti OR 'gut microflora':ab,ti OR 'microflora, gut':ab,ti OR 'gut microbiota':ab,ti OR 'gut microbiotas':ab,ti OR 'microbiota, gut':ab,ti OR 'gastrointestinal flora':ab,ti OR 'flora, gastrointestinal':ab,ti OR 'gut flora':ab,ti OR 'flora, gut':ab,ti OR 'gastrointestinal microbiota':ab,ti OR 'gastrointestinal microbiotas':ab,ti OR 'microbiota, gastrointestinal':ab,ti OR 'gastrointestinal microbial community':ab,ti OR 'gastrointestinal microbial communities':ab,ti OR 'microbial community, gastrointestinal':ab,ti OR 'gastrointestinal microflora':ab,ti OR 'microflora, gastrointestinal':ab,ti OR 'gastric microbiome':ab,ti OR 'gastric microbiomes':ab,ti OR 'microbiome, gastric':ab,ti OR 'intestinal microbiome':ab,ti OR 'intestinal microbiomes':ab,ti OR 'microbiome, intestinal':ab,ti OR 'intestinal microbiota':ab,ti OR 'intestinal microbiotas':ab,ti OR 'microbiota, intestinal':ab,ti OR 'intestinal microflora':ab,ti OR 'microflora, intestinal':ab,ti OR 'intestinal flora':ab,ti OR 'flora, intestinal':ab,ti OR 'enteric bacteria':ab,ti OR 'bacteria, enteric':ab,ti |  |
|  | **# 31: #28 OR #29 OR #30** | 108705 |
|  | **# 32: #27 AND #31** | 18866 |
|  | AND ('meta analysis'/de OR 'systematic review'/de) | 564 |
| **Epistemonikos** | **#1:** (title:(Nervous System Diseases) OR abstract:(Nervous System Diseases)) OR (title:(Neurocognitive Disorders) OR abstract:(Neurocognitive Disorders)) OR (title:(Central Nervous System) OR abstract:(Central Nervous System)) OR (title:(Mental Disorders) OR abstract:(Mental Disorders)) OR (title:(Brain Injuries) OR abstract:(Brain Injuries)) OR (title:(Parkinson Disease) OR abstract:(Parkinson Disease)) OR (title:(Multiple Sclerosis) OR abstract:(Multiple Sclerosis)) OR (title:(Alzheimer's disease) OR abstract:(Alzheimer's disease)) OR (title:(Brain Ischemia) OR abstract:(Brain Ischemia)) OR (title:(Intracranial Hemorrhages) OR abstract:(Intracranial Hemorrhages)) OR (title:(Hepatic Encephalopathy) OR abstract:(Hepatic Encephalopathy)) OR (title:(Stroke) OR abstract:(Stroke)) OR (title:(Spinal Cord Injuries) OR abstract:(Spinal Cord Injuries)) OR (title:(Traumatic Brain Injury) OR abstract:(Traumatic Brain Injury)) OR (title:(Amyotrophic Lateral Sclerosis) OR abstract:(Amyotrophic Lateral Sclerosis)) OR (title:(Epilepsy) OR abstract:(Epilepsy)) OR (title:(Autism Spectrum Disorder) OR abstract:(Autism Spectrum Disorder)) OR (title:(Attention Deficit Disorder with Hyperactivity) OR abstract:(Attention Deficit Disorder with Hyperactivity)) OR (title:(Hyperkinesis) OR abstract:(Hyperkinesis)) OR (title:(schizophrenia) OR abstract:(schizophrenia)) OR (title:(bipolar disorder) OR abstract:(bipolar disorder)) OR (title:(Cognitive Dysfunction) OR abstract:(Cognitive Dysfunction)) OR (title:(Alexithymia) OR abstract:(Alexithymia)) OR (title:(Depressive Disorder) OR abstract:(Depressive Disorder)) OR (title:(Dementia) OR abstract:(Dementia)) OR (title:(Anxiety Disorders) OR abstract:(Anxiety Disorders)) |  |
|  | **#2:** (title:(Gut-Brain Axis) OR abstract:(Gut-Brain Axis)) OR (title:(enteric nervous system) OR abstract:(enteric nervous system)) OR (title:(Gastrointestinal Microbiome) OR abstract:(Gastrointestinal Microbiome)) |  |
|  | **#3:** #1 AND #2 |  |
|  | Filters applied: Meta-Analysis, Systematic Review. | 54 |
